# Overexpression of a pseudo-etiolated-in-light-like protein in *Taraxacum koksaghyz* leads to a pale green phenotype and enables transcriptome-based network analysis of photomorphogenesis and isoprenoid biosynthesis

**DOI:** 10.3389/fpls.2023.1228961

**Published:** 2023-09-28

**Authors:** Silva Melissa Wolters, Vincent Alexander Benninghaus, Kai-Uwe Roelfs, Nicole van Deenen, Richard M. Twyman, Dirk Prüfer, Christian Schulze Gronover

**Affiliations:** ^1^ Fraunhofer Institute for Molecular Biology and Applied Ecology IME, Münster, Germany; ^2^ Institute for Biology and Biotechnology of Plants, University of Münster, Münster, Germany; ^3^ TRM Ltd, Scarborough, United Kingdom

**Keywords:** *Taraxacum*, chlorophyll biosynthesis, isoprenoids, light-dependent regulation, photomorphogenesis, pseudo-etiolation-in-light, RPGE

## Abstract

**Introduction:**

Plant growth and greening in response to light require the synthesis of photosynthetic pigments such as chlorophylls and carotenoids, which are derived from isoprenoid precursors. In *Arabidopsis*, the pseudo-etiolated-in-light phenotype is caused by the overexpression of *repressor of photosynthetic genes 2* (*RPGE2*), which regulates chlorophyll synthesis and photosynthetic genes.

**Methods:**

We investigated a homologous protein in the Russian dandelion (*Taraxacum koksaghyz*) to determine its influence on the rich isoprenoid network in this species, using a combination of *in silico* analysis, gene overexpression, transcriptomics and metabolic profiling.

**Results:**

Homology-based screening revealed a gene designated *pseudo-etiolated-in-light-like* (*TkPEL-like*), and *in silico* analysis identified a light-responsive G-box element in its promoter. *TkPEL-like* overexpression in dandelion plants and other systems reduced the levels of chlorophylls and carotenoids, but this was ameliorated by the mutation of one or both conserved cysteine residues. Comparative transcriptomics in dandelions overexpressing *TkPEL-like* showed that genes responsible for the synthesis of isoprenoid precursors and chlorophyll were downregulated, probably explaining the observed pale green leaf phenotype. In contrast, genes responsible for carotenoid synthesis were upregulated, possibly in response to feedback signaling. The evaluation of additional differentially expressed genes revealed interactions between pathways.

**Discussion:**

We propose that TkPEL-like negatively regulates chlorophyll- and photosynthesis-related genes in a light-dependent manner, which appears to be conserved across species. Our data will inform future studies addressing the regulation of leaf isoprenoid biosynthesis and photomorphogenesis and could be used in future breeding strategies to optimize selected plant isoprenoid profiles and generate suitable plant-based production platforms.

## 1 Introduction

Plants are photoautotrophic organisms that mostly depend on light for their energy supply. They also need to balance light exposure or mitigate excess irradiation to restrict photo-oxidative damage. Accordingly, they possess a complex network of light perception, signal transduction and effector proteins for the coordination of essential developmental processes such as photomorphogenesis and flowering (reviewed by Kami et al., 2010; Jing and Lin, 2020; Li et al., 2022). This starts with the perception of light signals by multiple families of photoreceptors that are translocated from the cytosol to the nucleus following activation (Genoud et al., 2008; Galvão and Fankhauser, 2015). In the nucleus, photoreceptors interact with E3 ubiquitin ligase complexes and transcription factors, which tune the activity of light-sensitive genes and thereby activate and coordinate downstream transcription cascades (Xu et al., 2014; Xu, 2019).

During photomorphogenesis, plants start to green and expand their leaves, requiring the synthesis of chlorophylls and other photosynthetic pigments. Chlorophyll and carotenoid biosynthesis requires isoprenoid precursors because the porphyrin ring of chlorophyll carries a phytol side chain and carotenoids are C_40_ isoprenoids (Cunningham and Gantt, 1998; Beale, 1999). Isoprenoids, also known as terpenoids, are one of the largest and most structurally diverse classes of natural products, consisting of many primary and secondary metabolites with various roles in basic cellular processes (Croteau et al., 2000; Tholl, 2015; Yazaki et al., 2017). The central metabolic precursors for the biosynthesis of all isoprenoids are the C_5_ isomers isopentenyl pyrophosphate (IPP) and dimethylallyl diphosphate (DMAPP), both of which can be synthesized via two metabolic routes: the cytosolic mevalonate (MVA) pathway and the plastidial methylerythritol phosphate (MEP) pathway (Vranová et al., 2012; Liao et al., 2016; Frank and Groll, 2017). The MVA pathway mainly supplies precursors for isoprenoids produced in the cytosol and mitochondria, such as sterols and brassinosteroids, whereas precursors produced by the MEP pathway form plastidial isoprenoids such as chlorophylls, carotenoids and plastoquinone (Lichtenthaler, 1999; Vranová et al., 2012). Both pathways are embedded in a tightly regulated, light-dependent network that is distributed over several cellular compartments. The pathways are regulated at transcriptional, post-transcriptional, translational and post-translational levels, and via feedback as well as feedforward signaling. This allows the metabolic flux to be allocated between different downstream pathways under normal and challenging conditions (reviewed in detail by Hemmerlin et al., 2012; Vranová et al., 2012; Tholl, 2015). However, the extent to which light perception/signaling and isoprenoid biosynthesis overlap is unclear and more work is required to understand how this complex network is regulated.

A systematic gain-of-function mutant screen in *Arabidopsis thaliana* revealed a line with pale green leaves and rapid development, a phenotype designated PEL meaning ‘pseudo-etiolation in light’ (Ichikawa et al., 2006). The gene overexpressed in this line encoded “plant protein 1589 of unknown function” (At3g55240). Given that all enzymes in the MEP and chlorophyll biosynthesis pathways are already known, the PEL gene was predicted to be a negative regulator (Beale, 1999; Vranová et al., 2012). The overexpression phenotype was confirmed in a later study and the gene was named *repressor of photosynthetic genes 2* (*RPGE2*) based on the identification of its paralog *RPGE1* as an early red-light-responsive gene regulated by phytochrome interacting factors (PIFs), and the repression of photosynthetic genes in *RPGE1* and *RPGE2* overexpression lines (Leivar et al., 2009; Zhang et al., 2013; Kim K. et al., 2016). PIFs have been described as light-dependent negative transcriptional regulators of photomorphogenesis and the MEP pathway (Chenge-Espinosa et al., 2018), as well as chlorophyll and carotenoid biosynthesis (Huq et al., 2004; Moon et al., 2008; Toledo-Ortiz et al., 2010; Tang et al., 2012; Job and Datta, 2021). They are potential cofactors of COP1 (constitutive photomorphogenic 1), an E3 ubiquitin ligase that represses photomorphogenesis in the dark by accumulating in the nucleus and mediating the degradation of light-dependent positive regulators such as the bZIP transcription factor Long hypocotyl 5 (HY5) (Deng et al., 1991; Osterlund et al., 1999; Saijo et al., 2003; Jang et al., 2005; Xu et al., 2014). Very recent findings in *Arabidopsis* have shown that AtRPGE1 and AtRPGE2 suppress chlorophyll biosynthesis by inhibiting the transcription factor Golden2-like 1 (GLK1) (Kim et al., 2023), which activates genes involved in chlorophyll biosynthesis, light harvesting and electron transport (Waters et al., 2009).

Comparative transcriptomics in carrots (*Daucus carota*) with high and low pigment levels revealed an association between carotenoid accumulation in roots with one or two recessive mutations in the gene *DCAR_032551*, which is homologous to *AtRPGE2*, expanding the function of this protein to root tissues and carotenoid synthesis (Iorizzo et al., 2016). Furthermore, multiple light-induced genes and genes involved in the MEP pathway and carotenoid biosynthesis are upregulated in strongly pigmented carrots (Iorizzo et al., 2016). The function of the DCAR_032551 protein is unclear, but it was proposed to regulate carotenoid accumulation resulting from de-etiolation in roots that have lost the ability to repress the transcriptomic cascade connected to photomorphogenesis (Iorizzo et al., 2016).

The *AtRPGE2* and *DCAR_032551* genes encode interesting regulatory proteins in the light signaling network affecting isoprenoid metabolism, and the analysis of orthologs in other species could provide more insight into their mode of action and evolutionary origin. A plant ideally suited to analyze photomorphogenic isoprenoid metabolism is the Russian dandelion (*Taraxacum koksaghyz*), because it produces high levels of various isoprenoid compounds in different tissues. It is also a valuable source of economically important isoprenoids (van Beilen and Poirier, 2007), in particular due to the large amounts of high-quality natural rubber formed in the latex of its roots (Ulmann, 1951; van Beilen and Poirier, 2007; Ramirez-Cadavid et al., 2017). Research has focused on domesticating this wild plant to make a profitable and sustainable rubber crop, especially by investigating the transcriptomic, proteomic and metabolomic properties of its roots (Niephaus et al., 2019; Pütter et al., 2019; Benninghaus et al., 2020). Only recently has the scope of this work expanded to include the transcriptomic and metabolic profiles of the leaves (Cheng et al., 2022; Panara et al., 2022; Zhang et al., 2022).

To broaden our knowledge of the rich isoprenoid metabolic network in the Russian dandelion, we screened for homologs of AtRPGE2/DCAR_032551 to investigate their regulatory functions. We identified the *T. koksaghyz* gene *pseudo-etiolated-in-light-like* (*TkPEL-like*), which like its orthologs in *Arabidopsis* and carrot reduced leaf chlorophyll and carotenoid levels when overexpressed. The function of TkPEL-like depends on two conserved cysteine residues. Comparative transcriptomics in dandelions overexpressing *TkPEL-like* vs controls provided information about affected pathways and their interactions, and highlighted the suggested conservation of these properties across species.

## 2 Materials and methods

### 2.1 Cloning of *TkPEL-like* and vector construction

The TkPEL-like coding sequence was amplified by PCR from *T. koksaghyz* cDNA using primers TkPEL-like_fw and TkPEL-like_rev (**Supplementary Table S1**). It was purified using the PCR clean-up gel extraction kit (Macherey-Nagel, Germany) and inserted into the cloning vector pJET1.2/blunt using the CloneJET PCR Cloning Kit (Thermo Fisher Scientific, USA). For the *TkPEL-like* overexpression construct, the strong quadruple cauliflower mosaic virus promoter (pQ35S) was amplified from pFGC5941-GW (GenBank: DQ231581.1) using primers Q35S-P_XmaI_fw and Q35S-P_SOE_rev_XhoI_mut, and the TMV Ω 5′-leader sequence was amplified from the same plasmid using primers TMV_Omega_TL_SOE_fw_XhoI_mut and TMV_Omega_TL_XhoI_rev. The PCR products were diluted 1:100 and used as templates for overlap extension PCR with primers Q35S-P_XmaI_fw and TMV_Omega_TL_XhoI_rev. This disrupted the XhoI site between pQ35S and the TMV Ω 5′-leader, enabling the subsequent cloning step. The PCR product was purified and digested (XmaI + XhoI) and inserted into pLab12.1 (Post et al., 2012), which had been digested with the same enzymes. The *TkPEL-like* sequence was amplified from pJET-*TkPEL-like* using primers TkPEL-like_NcoI_fw and TkPEL-like_XhoI_rev. After purification and digestion (XhoI + XbaI), the *TkPEL-like* sequence was inserted into the XhoI/XbaI-digested custom vector for constitutive expression. For transient expression in *Nicotiana benthamiana*, the *TkPEL-like* sequence was amplified from pJET-*TkPEL-like* using forward primer TkPEL-like_NcoI_fw and either TkPEL-like_XhoI_rev or TkPEL-like_XhoI_rev_nsc, yielding fragments with and without a stop codon. These fragments were purified, digested (NcoI + XhoI) and inserted into the NcoI/XhoI-digested Gateway vector pENTR4 (Invitrogen, USA), resulting in the cloning vectors pENTR4-*TkPEL-like* and pENTR4-*TkPEL-like*_NSC (NSC = no stop codon). Site-directed mutagenesis was carried out with primer pairs TkPEL-like_muta.t55a and TkPEL-like_muta.t55a_antis or TkPEL-like_muta.t85a and TkPEL-like_muta.t85a_antis using the QuikChange Lightning Site-Directed Mutagenesis Kit and the publicly available QuikChange Primer Design Program (Agilent Technologies, USA), leading to the nucleotide substitutions *c.*55T>A and/or *c.*85T>A. This yielded the following cloning vectors: pENTR4-*TkPEL-like*_t55a, pENTR4-*TkPEL-like*_t85a, pENTR4-*TkPEL-like*_t55a/t85a, pENTR4-TkPEL-like_NSC_t55a, pENTR4-*TkPEL-like*_NSC_t85a and pENTR4-*TkPEL-like*_NSC_t55a/t85a. Finally, the mutated and non-mutated sequences were transferred to the Gateway-compatible vectors pBatTL-*ccdB*, pBatTL-*Cerulean-ccdB* and pBatTL-*ccdB-Cerulean* (Epping et al., 2015) by LR recombination using LR Clonase (Thermo Fisher Scientific), resulting in the final plant transformation vectors pBatTL-*TkPEL-like_t55a*, pBatTL-*TkPEL-like_t85a*, pBatTL-*TkPEL-like_t55a/t85a*, pBatTL-*Cerulean-TkPEL-like_t55a*, pBatTL-*Cerulean-TkPEL-like_t85a*, pBatTL-*Cerulean-TkPEL-like_t55a/t85a*, pBatTL-*TkPEL-like_t55a-Cerulean*, pBatTL-*TkPEL-like_t85a-Cerulean* and pBatTL-*TkPEL-like_t55a/t85a-Cerulean*. All constructs were sequenced to confirm their integrity.

### 2.2 Plant cultivation and tissue processing

All *T. koksaghyz* plants were cultivated under controlled conditions in an indoor greenhouse (18°C, 16‐h photoperiod, 260 PPFD high-pressure sodium lamp with enhanced yellow and red spectrum) as previously described (Unland et al., 2018). Plant tissues were separated during harvesting, then immediately flash-frozen in liquid nitrogen and lyophilized. The root tissue was pulverized using a ZM 200 Ultra Centrifugal Mill (Retsch, Germany), whereas petiole, leaf and flower tissues were pulverized under liquid nitrogen with mortar and pestle.

### 2.3 Generation of pQ35S::*TkPEL-like T. koksaghyz* plants and transgene verification

The *TkPEL-like* overexpression construct was introduced into *T. koksaghyz* plants (accession number 203; kindly provided by the Botanical Garden of the University of Münster, Germany) as previously described (Stolze et al., 2017). The resulting transgenic plants were selected by cultivation on phosphinothricin-containing medium. The presence of the transgenes was verified in crude leaf extracts by PCR with gene-specific primers (**Supplementary Table S1**) using the KAPA3G Plant PCR Kit (Kapa Biosystems, USA).

### 2.4 RNA extraction and cDNA synthesis

Total RNA was extracted from *T. koksaghyz* root, latex, leaf, petiole and flower tissues using the innuPREP RNA Mini Kit (Analytik Jena, Germany) according to the manufacturer’s instructions. To synthesize full-length cDNA, 500 ng of total RNA was reverse transcribed using PrimeScript RT Master Mix (TAKARA Clontech, USA) according to the manufacturer’s instructions.

### 2.5 Gene expression analysis by qRT-PCR

Endogenous spatiotemporal expression patterns were determined by quantitative real-time PCR (qRT-PCR) as previously described (Laibach et al., 2015) using RNA from the root, latex, petiole, leaf without midrib and flower tissues of 12-week-old *T. koksaghyz* wild-type plants grown outdoors from May to August 2017 (University of Münster, Germany) or leaf RNA derived from wild-type plants aged 3, 4, 5, 6, 8, 10 or 12 weeks grown under controlled greenhouse conditions as above. For the spatial expression pattern, three pools consisting of four individual plants as biological replicates were tested for each tissue. For the temporal expression pattern, three independent wild-type plants as biological replicates were tested for each time point. *TkPEL-like* overexpression in transgenic plants was assessed for eight individual plants from each of the lines #3.4, #4.1 and #5.7 in comparison to nine individual plants of near-isogenic lines (NILs). For the validation of the transcriptomic data, leaf RNA from three pools each consisting of four plants was analyzed for the transgenic lines #3.4, #4.1 and #5.7. In comparison, three pools consisting of leaf RNA from two to three plants each was analyzed for the corresponding NILs (NIL#3.4, NIL #4.1, NIL #5.7). All samples were additionally analyzed in three technical replicates. We used *elongation factor 1a* (*TkEf1a*) and *ribosomal protein L27* (*TkRP*) to calculate the relative normalized expression levels (Pütter et al., 2017) using Bio-Rad CFX Manager v3.1 (Bio-Rad Laboratories, USA). All oligonucleotides are listed in **Supplementary Table S1**.

### 2.6 Infiltration of *N. benthamiana* leaves and microscopy

Transient expression in 4-week-old *N. benthamiana* leaves was achieved by agroinfiltration (Müller et al., 2010). *N. benthamiana* seeds (kindly provided by the Sainsbury Laboratory, John Innes Centre, Norwich, UK) were germinated and the plants were cultivated under controlled greenhouse conditions (Unland et al., 2018). After infiltration, the plants were grown for 4–5 days under steady light in a growth chamber (CLF Plant Climatics, Germany) at 19°C. The subcellular localization of recombinant proteins in epidermal cells was determined by screening tissue explants from infiltrated leaves for Cerulean fluorescence using the Leica TCS SP5 X confocal system (Leica Microsystems, Germany) with excitation at 405/458 nm and emission at 469–488 nm.

### 2.7 Quantification of carotenoids and chlorophylls in leaves

Leaf material was harvested, immediately flash-frozen in liquid nitrogen, lyophilized in a freeze dryer Alpha 1-4 LSCplus (Christ Gefriertrocknungsanlagen, Germany) and cryogenically ground in a Retsch mixer mill MM 400. We then extracted 10 mg of the pulverized leaf powder twice with 500 µl acetone (30 min, room temperature, on a shaking platform in the dark) and removed the cell debris by centrifugation (1000 g, 5 min, room temperature). The final 1 ml acetone leaf extract was diluted 1:10 with acetone and analyzed by absorption spectrophotometry at 470, 645, 662 and 750 nm using a quartz cuvette and a BioSpectrometer (Eppendorf, Germany). The carotenoid and chlorophyll contents were calculated as previously described (Lichtenthaler, 1987).

### 2.8 Quantification of root metabolites

Poly(*cis*-1,4-isoprene), squalene/2,3-oxidosqualene and pentacyclic triterpenes/triterpenoids were measured in pulverized root extracts by ¹H-NMR and GC-MS (Stolze et al., 2017).

### 2.9 Chlorophyll fluorescence measurements

The maximum potential quantum efficiency of photosystem II (PSII) and non-photochemical quenching (NPQ) were measured in a single leaf from 12-week-old transgenic *T. koksaghyz* plants and controls using a Maxi-Imaging Pulse-Amplitude-Modulation (PAM) chlorophyll fluorimeter (Walz, Germany). Before measurement, the leaves were dark adapted for 20 min, exposed to photosynthetically active radiation with an intensity of 1076 μmol m^-2^ s^-1^ for 5 min, followed by relaxation in the dark for ~15 min. Fluorescence measurements were captured from 14 independent areas per leaf. The photosynthetic parameters, described in detail by Baker (2008), were calculated using ImagingWin v2.41a (Walz). NPQ was calculated using Equation 1, the maximum quantum yield of PSII after dark adaption (F_v_/F_m_) using Equation 2, and the operating efficiency of PSII under illumination (F_q_′/F_m_′ or Φ_PSII_) using Equation 3.

Equation 1: $NPQ=\frac{Fm-Fm'}{Fm'}$

Equation 2: $\frac{F_{v}}{F_{m}}=\frac{F_{m}-F0}{F_{m}}$

Equation 3: $\frac{F_{q}^{'}}{F_{m}^{'}}=\frac{F_{m}^{'}-F'}{F_{m}^{'}}$ ,

### 2.10 *In silico* analysis

The detection of distant *TkPEL-like* homologs was achieved by screening the non-redundant NCBI peptide database (NR, http://www.ncbi.nlm.nih.gov). Multiple sequence alignment was then carried out using Clustal Omega (https://www.ebi.ac.uk/Tools/msa/clustalo/) and MegAlign Pro 17 (DNASTAR, USA). MOTIF (https://www.genome.jp/tools/motif/) was used to screen the NCBI-CDD library (Marchler-Bauer et al., 2013), as well as the Pfam (Bateman et al., 2002) and PROSITE (Falquet et al., 2002) databases for matching protein domains. Evolutionarily conserved amino acids were visualized using the ConSurf server (http://consurf.tau.ac.il; Ashkenazy et al., 2016), and secondary structures were predicted using the JPred 4 server (Drozdetskiy et al., 2015). The subcellular localization of proteins was predicted using DeepLoc (Armenteros et al., 2017; Armenteros et al., 2019). The *TkPEL-like* promoter was analyzed using NSITE-PL (Shahmuradov and Solovyev, 2015).

### 2.11 Transcriptomic comparison

RNA was extracted from the leaf material of four plants from each pQ35S::*TkPEL-like* line (#3.4, #4.1 and #5.7) and four plants of the corresponding NILs. The RNA obtained from four individual plants belonging to one line was pooled in equal amounts. The three RNA pools per genotype were then pooled again in equal amounts resulting in two RNA pools consisting of 12 plants each that were used for Illumina 150-bp paired-end sequencing (Novogene, USA). Reads were mapped to the *T. koksaghyz* reference genome (Lin et al., 2022) using Subread v2.0.3 and raw counts were determined using Feature Counts. Transcripts were annotated by conducting a BLASTX search against the NCBI NR and UniProt databases with a minimum e-value of 1 × 10^-3^. Differential gene expression was analyzed using DEGseq v1.50.0. Datasets were filtered for minimum counts per million (CPM) of 0.2 and differential expression was assessed by comparing CPMs in NILs and pQ35S::*TkPEL-like* plants. Differentially expressed genes (DEGs) were accepted if they met the threshold for log_2_ fold changes – log_2_(FC) – in normalized expression ≥ 1 or ≤ –1 and a q-value (Storey) < 0.05 (**Supplementary Data S1**). Gene Ontology (GO) term enrichment among modulated genes (–2.32 ≤ log_2_(FC) ≥ 2.32) was calculated using WEGO (https://wego.genomics.cn/) with all transcripts detected in NILs as the background. Genes were also annotated against the Kyoto Encyclopedia of Genes and Genomes (KEGG) database using eggNOG-mapper v2 (http://eggnog-mapper.embl.de/). KEGG pathway enrichment analysis was carried out using the enricher tool of the clusterProfiler package in R Studio (Posit PBC, USA).

## 3 Results

### 3.1 Identification and *in silico* characterization of *TkPEL-like*

**Figure 1:**
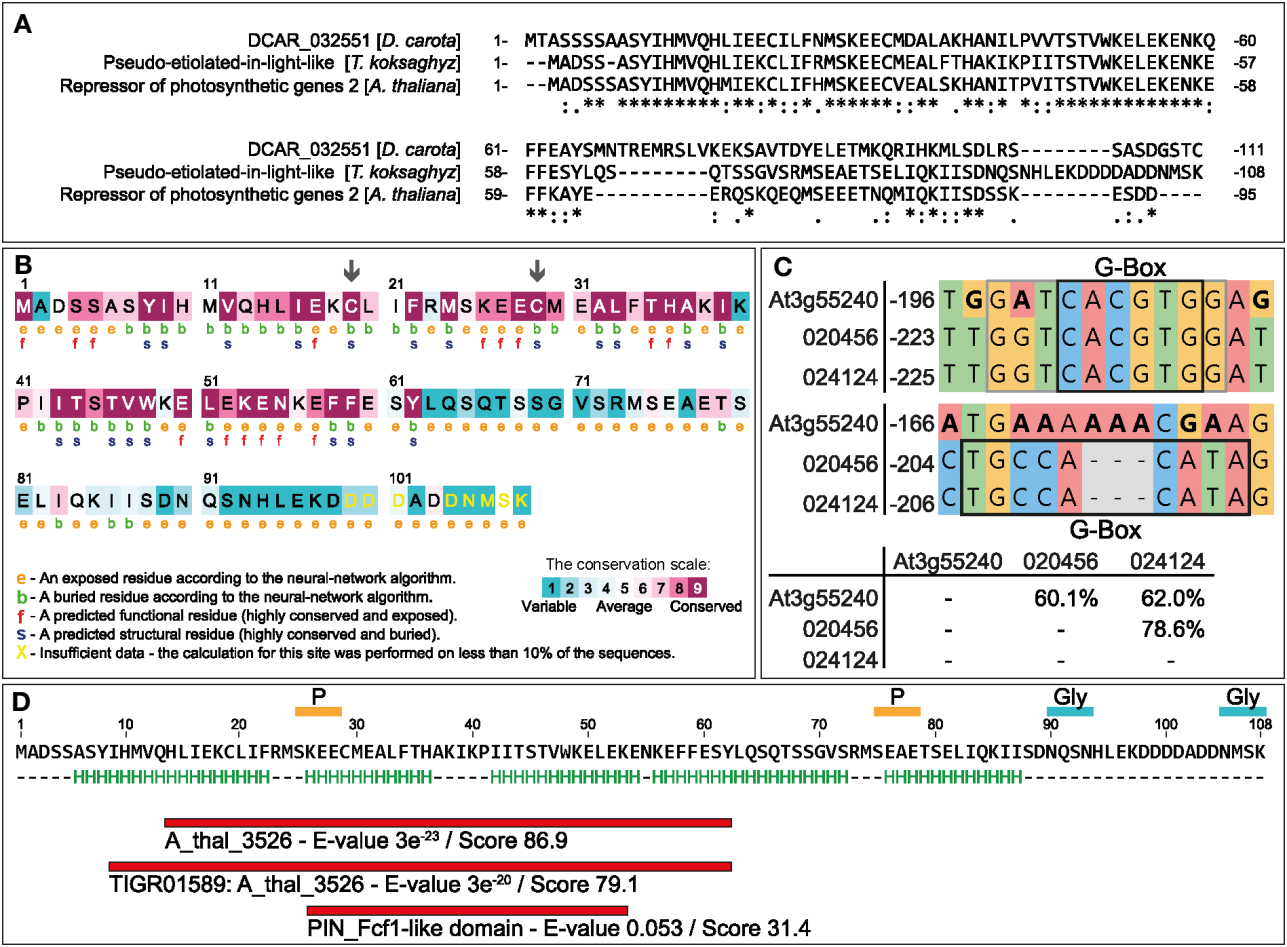
*In silico* analysis of the TkPEL-like protein and the corresponding promoter. **(A)** Multiple sequence alignment of TkPEL-like, DCAR_032551 from *D. carota* (GenBank: KZM94650.1) and AtRPGE2 (At3g55240). * (asterisk) indicates positions which have a single, fully conserved residue; : (colon) indicates conservation between groups of strongly similar properties - roughly equivalent to scoring > 0.5 in the Gonnet PAM 250 matrix; . (period) indicates conservation between groups of weakly similar properties - roughly equivalent to scoring ≤ 0.5 and > 0 in the Gonnet PAM 250 matrix. **(B)** Identification of functional and conserved regions within the TkPEL-like protein using ConSurf based on multiple sequence alignments. **(C)** Promoter sequence comparisons between two *TkPEL-like* loci in *T. koksaghyz* and *AtRPGE2* based on multiple sequence alignments. IDs refer to gene IDs of the *T. koksaghyz* genome (Lin et al., 2022). Black boxes indicate G-box core sequences. Gray box indicates variable G-box extensions. **(D)** TkPEL-like amino acid sequence with proposed helix structures (H) and predicted conserved protein domains identified by Motiffinder.

A homology search against *T. koksaghyz* sequence databases identified Contig19977 (length 751 bp, e-value 2 × 10^-29^) with a translated sequence similar to DCAR_032551. Amplification with flanking primers yielded a shorter, 327-bp coding sequence, which we named *TkPEL-like*. A subsequent, less stringent BLASTP search with the obtained 108 aa sequence identified homologous protein sequences to be most prominent in clade Streptophyta (**Supplementary Figure S1**). They included only a few annotated proteins: a DNA replication complex GINS protein, a putative angiotensin-converting enzyme 2 and a polyribonucleotide nucleotidyltransferase. Nucleotide-based homology searching revealed an additional match to an argininosuccinate lyase (**Supplementary Table S2**). Multiple sequence alignment of the TkPEL-like protein, DCAR_032551 and AtRPGE2 showed that the 57 N-terminal amino acids are strongly conserved (**Figure 1A**), with three overlapping domains: ‘A_thal_3526’ (CDD: 401595) and ‘TIGR01589: A_thal_3526’ (CDD: 130650) are only found in plant proteins but their functions are unknown, whereas the PIN_Fcf1-like domain (CDD: 350212) is present in a large nuclease superfamily involved in RNA processing (**Figure 1D**). The N-terminal sequence also contains two highly conserved cysteines that are predicted to act as structural residues (arrows in **Figure 1B**). Secondary structure prediction revealed five potential α-helices (H, marked in green) distributed throughout the peptide sequence (**Figure 1D**). Additionally, the protein contains two putative *N*-glycan accepter sites (AA90-93, 105-108) and two phosphorylation sites (AA25-28,75-78). Like its carrot and *Arabidopsis* counterparts, TkPEL-like was predicted to be a cytosolic protein (**Supplementary Figure S1**). Given that the *AtRPGE2* promoter contains sequences recognized by PIFs (Leivar et al., 2009; Zhang et al., 2013; Kim K. et al., 2016) and the bZIP transcription factor HY5, a key regulator of light-mediated transcriptional programming (Lee et al., 2007), we also analyzed the *TkPEL-like* promoter for the corresponding binding sites. First, we searched the *T. koksaghyz* genome assembly (Lin et al., 2022) for homology to the 327-bp amplified gene sequence to identify the promoter region, revealing two matching loci with 95–99% identity. We then screened 1 kb upstream of each coding sequence to identify *cis*-acting regulatory motifs. We found 20 motifs for GWHGBCHF020456 on pseudochromosome four, and 21 for GWHGBCHF024124 on pseudochromosome five, including a G-box motif with the core sequence CACGTG. This is known to mediate light-responsive transcriptional regulation by binding to HY5 and PIFs (Toledo-Ortiz et al., 2003; Lee et al., 2007; Kim J. et al., 2016) (**Figure 1C**; **Supplementary Table S3**). This motif also shows similarity to the light-dependent regulatory element ACE^CHS^, which is present in the *chalcone synthase* (*CHS*) promoter and bound by the bZIP transcription factors CPRF1 and CPRF4 to regulate flavonoid biosynthesis (Kircher et al., 1998; Sprenger-Haussels and Weisshaar, 2000).

### 3.2 *TkPEL-like* is predominantly expressed in the leaves of adult *T. koksaghyz* plants

**Figure 2:**
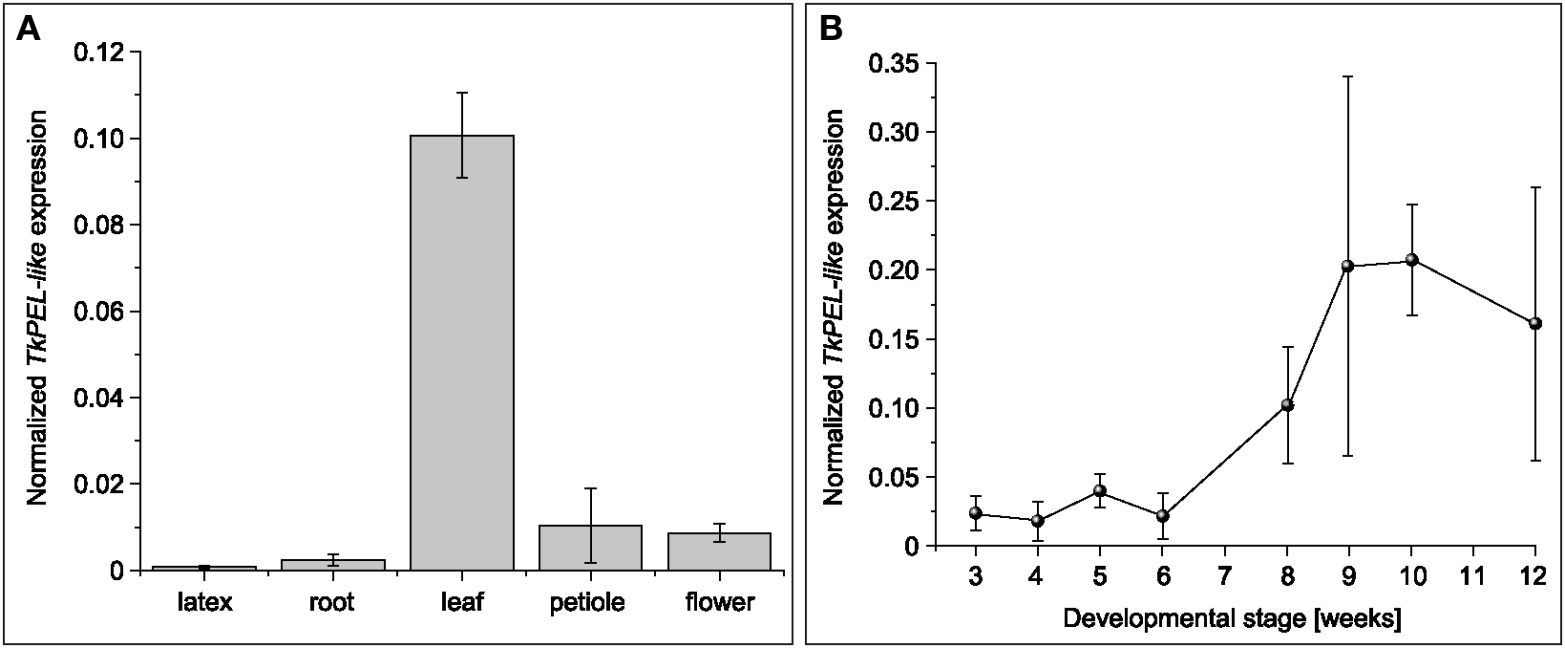
*TkPEL-like* is predominantly expressed in the mature leaves of wild-type *T. koksaghyz* plants. **(A)** Normalized transcript levels of *TkPEL*-like in different tissues of wild-type *T. koksaghyz*. Data are means (± SD) of three pools of four individual plants. **(B)** Time course of *TkPEL-like* expression in wild-type *T. koksaghyz* leaves. Plants were grown under controlled greenhouse conditions. Expression levels were normalized against *elongation factor 1a* (*TkEf1a*) and *ribosomal protein L27* (*TkRP*). Data are means (± SD) of three independent plants.

To examine the spatial expression profile of *TkPEL-like*, we used cDNA obtained from latex, root, leaf, petiole and flower tissues of 12-week-old *T. koksaghyz* plants for qRT-PCR analysis. This revealed predominant expression in leaves (**Figure 2A**). We then evaluated the temporal expression profile of *TkPEL-like* in leaves, which indicated low levels of gene expression during the growth stages (weeks 3–6) followed by a ~5-fold increase in weeks 8–12 (**Figure 2B**).

### 3.3 Heterologous expression of *TkPEL-like* in *N. benthamiana* reveals cytosolic and nuclear protein localization and reduced levels of chlorophylls and carotenoids

**Figure 3:**
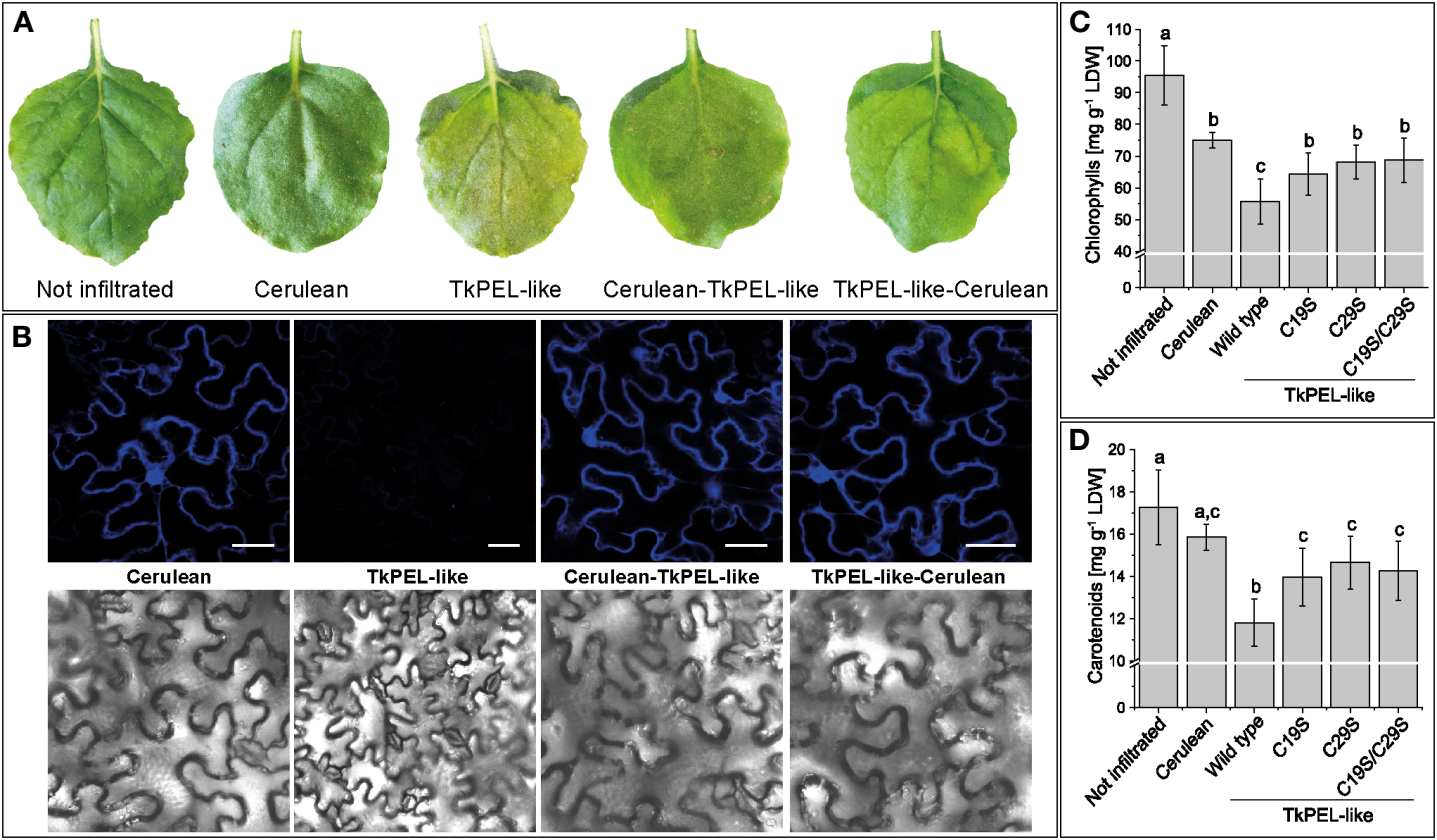
Heterologous expression of *TkPEL-like* in *N. benthamiana* leaf epidermal cells results in cytosolic and nuclear protein localization and reduced carotenoid and chlorophyll levels. **(A)** Phenotype of leaves expressing N-terminal and C-terminal TkPEL-like Cerulean fusions and corresponding controls. Leaves expressing *TkPEL-like* appear light green. **(B)** Confocal laser scanning microscopy images of leaves expressing TkPEL-like Cerulean fusions and corresponding controls. Scale bar = 40 µm. **(C, D)** Chlorophyll and carotenoid levels in leaves expressing wild-type *TkPEL-like* and three different mutant versions lacking conserved cysteine residues at position 19 and/or 29 compared to *Cerulean-*expressing and non-infiltrated controls. The chlorophyll content was calculated based on chlorophyll *a* and *b* (**Supplementary Table S3**). Data are means (± SD) of four independently infiltrated leaves per construct. Significant differences were assessed by ANOVA with Tukey’s honest significant difference test (p < 0.05). The lower case letters represent the statistically different groups.

We generated expression constructs in which the fluorophore Cerulean was fused in-frame with the TkPEL-like coding sequence at the N-terminus or C-terminus in order to investigate its intracellular localization *in vivo*. Transient expression in *N. benthamiana* leaf epidermal cells was monitored by confocal laser scanning microscopy, showing Cerulean fluorescence signals in the cytosol and nucleus for TkPEL-like fusions as well as Cerulean itself (**Figure 3B**). This suggests TkPEL-like is localized in these compartments, although we cannot exclude the possibility that the spatial distribution was due to the small size of the fusion protein. Leaves transiently expressing native *TkPEL-like* or the *Cerulean* fusions showed visible signs of chlorosis after 4 days (**Figure 3A**). We therefore measured the pigment content of the leaves and compared them with non-infiltrated leaves and those infiltrated with a *Cerulean* construct alone. We found that chlorophyll and carotenoid levels were significantly lower in leaves expressing *TkPEL-like* constructs compared to controls (**Figures 3C, D**; **Supplementary Table S4**).

To assess the role of the two conserved cysteine residues in the highly conserved N-terminus, we introduced either single mutations (C19S or C29S) or a double mutation (C19S, C29S) in the fusion constructs. Microscopy revealed comparable cytosolic and nuclear fluorescence for the mutants and the wild-type variant (**Supplementary Figure S2**). This was supported by *in silico* secondary structure predictions, which showed that the five α-helices were preserved in the mutants, confirming no significant impact on the integrity of the protein’s structure (**Supplementary Figure S3**). Pigment analysis of leaves expressing one of the three mutant *TkPEL-like* sequences alone or as *Cerulean* fusions again showed a reduction in chlorophyll and carotenoid levels (**Figures 3C, D**; **Supplementary Table S4**). However, the amounts were comparable to those detected in leaves expressing *Cerulean* alone. Accordingly, leaves infiltrated with the *TkPEL-like* wild-type constructs had significantly lower levels of photosynthetic pigments than leaves expressing the mutant variants or *Cerulean*, suggesting the conserved cysteine residues are important for the function of TkPEL-like.

### 3.4 Overexpression of *TkPEL-like* in *T. koksaghyz* causes a pale green phenotype

**Figure 4:**
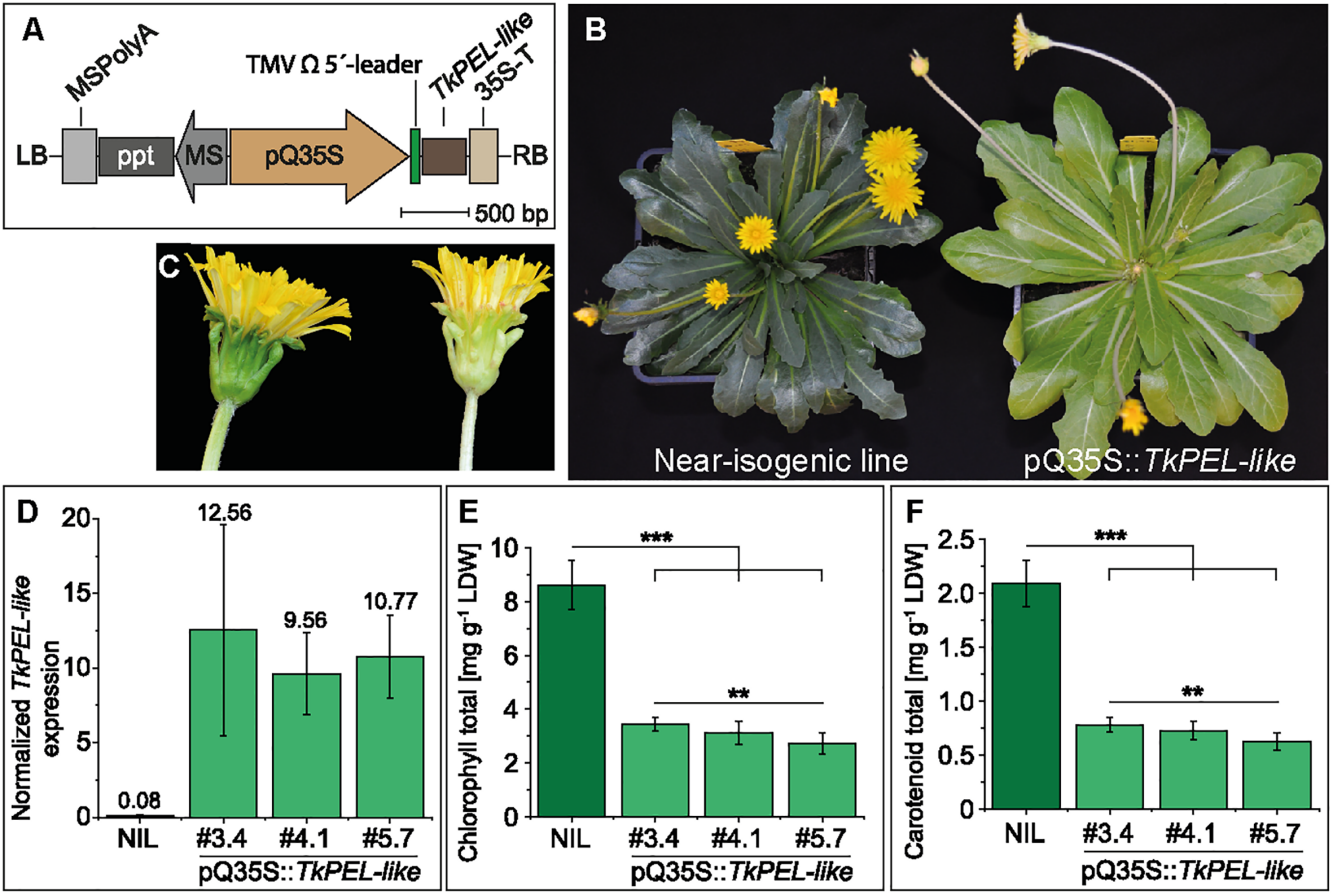
Generation and characterization of pale green *T. koksaghyz* plants overexpressing *TkPEL-like.* **(A)** Schematic representation of the T-DNA carrying the pQ35S promoter and TMV Ω 5′-leader sequence to achieve strong, constitutive expression of the *TkPEL-like* coding sequence in plants. Resistance gene cassettes: MS, *mannopine synthase* promoter; ppt, phosphinothricin resistance gene; MSPolyA, poly(A) sequences from the mannopine synthase gene. **(B)** 12-week-old T_1_ generation plants of a near isogenic line (NIL) and a *TkPEL-like* overexpression line pQ35S::*TkPEL-like*. **(C)** Close-up of flowers from the NIL (left) and pQ35S::*TkPEL-like* (right) representing the T_1_ generation. **(D-F)** Analysis of nine individual NIL plants and eight individual plants as biological replicates from three independent pQ35S::*TkPEL-like* lines (#3.4, #4.1 and #5.7) of the T_2_ generation grown under controlled greenhouse conditions for 12 weeks. **(D)** Normalized expression levels of *TkPEL-like* confirm overexpression in leaves of pQ35S::*TkPEL-like* lines. Expression levels were normalized against *elongation factor 1a* (*TkEf1a*) and *ribosomal protein L27* (*TkRP*). Numbers above SD error bars are mean values. **(E)** Leaf chlorophyll content based on chlorophyll *a* and *b* (**Supplemental Table S4**). **(F)** Leaf carotenoid levels. Statistical differences in chlorophyll and carotenoid contents were assessed using non-parametric Mann-Whitney U-tests (**p < 0.01, ***p < 0.001).

The ubiquitous overexpression of *TkPEL-like* in *T. koksaghyz* was achieved by placing the gene under the control of the pQ35S promoter (**Figure 4A**). Several transgenic lines were generated and crossed with wild-type *T. koksaghyz* to obtain the T_1_ generation. The resulting progeny showed two alternative genotypes: transgenic plants that carried the *TkPEL-like* overexpression construct, which were named pQ35S::*TkPEL-like*, and NILs lacking the transgene. In common with the T_0_ generation, the T_1_ generation of pQ35S::*TkPEL-like* plants showed a pale green leaf phenotype and a white midrib (**Figure 4B**). The normally green parts of the petioles and flowers also appeared whitish (**Figures 4B, C**).

For in-depth analysis, we used three independent transgenic lines (#3.4, #4.1 and #5.7) grown from seeds harvested from T_1_ plants crossed with wild-type plants, thus representing the T_2_ generation. The resulting pQ35S::*TkPEL-like* plants showed no abnormal morphology (**Supplementary Figure S4**) or differences in growth rate, size of the aboveground tissues or flowering (**Supplementary Figure S5**) compared to NIL controls, but still had a pale green/whitish appearance, which confirmed the stable inheritance of this phenotype. Next, we used leaf cDNA for qRT-PCR analysis, which confirmed a massive increase in *TkPEL-like* expression (137-fold, on average) in the pQ35S::*TkPEL-like* plants compared to NIL controls (**Figure 4D**). We also observed a significant reduction in the amount of chlorophylls and carotenoids in the transgenic leaves compared to NIL leaves (**Figures 4E, F** and **Supplementary Table S5**), explaining the observed phenotype. After observing these effects on leaf isoprenoids, we tested the roots of the pQ35S::*TkPEL-like* plants because *T. koksaghyz* roots normally accumulate large quantities of isoprenoid end-products such as pentacyclic triterpenes and poly(*cis*-1,4-isoprene), the main component of natural rubber (Niephaus et al., 2019; Pütter et al., 2019). *TkPEL-like* overexpression was confirmed in the roots of all three transgenic lines, but the quantity of pentacyclic triterpenes and poly(*cis*-1,4-isoprene) remained similar to normal (**Supplementary Figure S6**; **Supplementary Table S6**). However, the dry root weight of lines #3.4 and #5.7 was significantly lower than that of NIL controls (**Supplementary Figure S6**).

### 3.5 The maximum quantum yield of PSII increases in pQ35S::*TkPEL-like* plants

**Figure 5:**
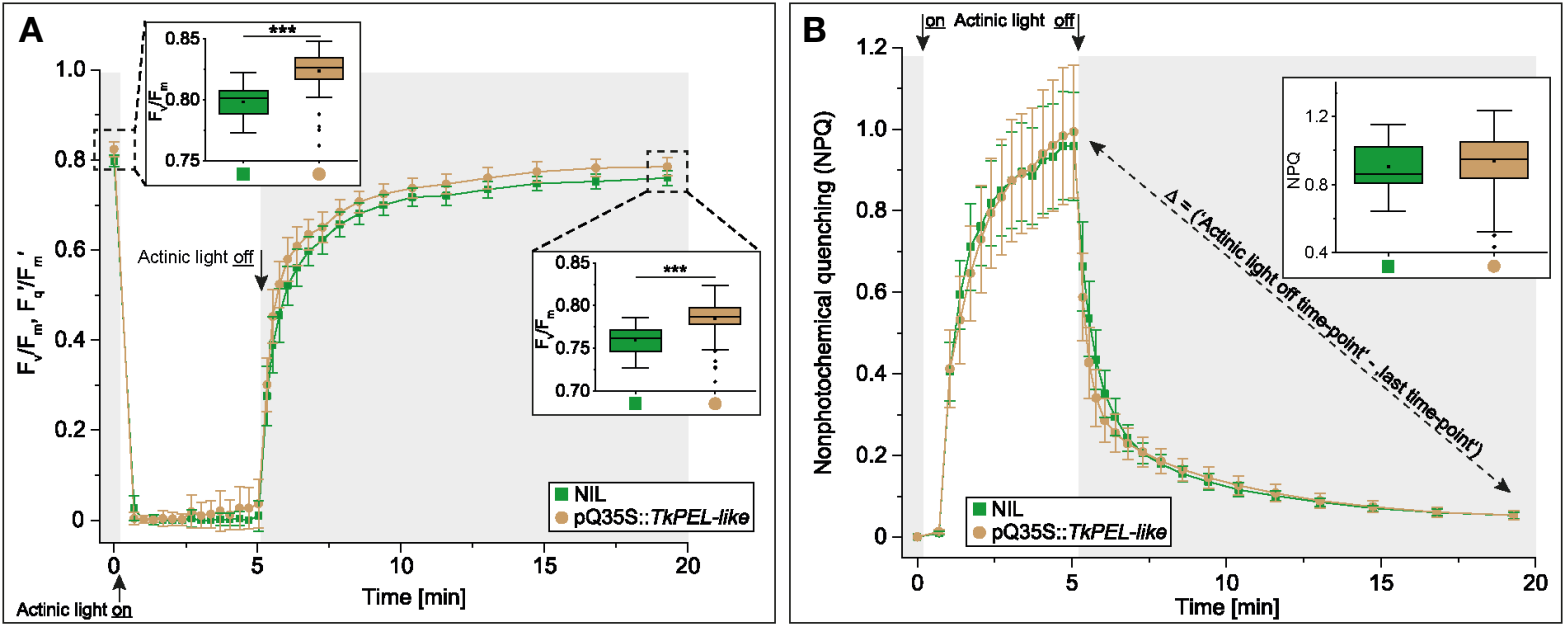
Quantum efficiency of photosystem II increases in 12-week-old pQ35S::*TkPEL-like* plants but non-photochemical quenching is not affected. **(A)** Measurement of photosystem II (PSII) maximum (F_v_/F_m_) and effective (F_q_′/F_m_′) quantum yield and **(B)** Measurement of non-photochemical quenching (NPQ) following transition from dark to intense light. Data are means (± SD) of 13 pQ35S::*TkPEL-like* plants and 13 NILs. One leaf from each plant was harvested for measurement. Leaves were dark adapted for 20 min before applying photosynthetically active radiation (actinic light) with an intensity of 1076 μmol quanta m^-2^ s^-1^ for 5 min. This was followed by relaxation in the dark for ~15 min. The fluorescence levels of 14 independent areas per leaf were measured. The framed bar charts in panel A depict the first (left) and last (right) data points of the measurement. The framed bar chart in panel B depicts the calculated difference in NPQ of the data point ‘actinic light off’ and the last data point. Statistical differences were assessed using non-parametric Mann-Whitney U-tests (***p < 0.001).

To compare the photosynthetic performance of wild-type and pale green leaves, we measured the leaf fluorescence of 12-week-old NIL and pQ35S::*TkPEL-like* plants. We measured the maximum quantum yield (F_v_/F_m_) and the effective quantum yield (F_q_′/F_m_′) of PSII in three consecutive periods: dark-adaption, exposure to intense light, and during recovery in darkness (**Figure 5A**). During short-term illumination, both genotypes showed stable and comparably low F_q_′/F_m_′ values. In contrast, the F_v_/F_m_ values of both genotypes in the absence of light appeared to match those of leaves that were typically non-stressed (Björkman and Demmig, 1987), but this value was consistently and significantly higher for the pale green leaves of pQ35S::*TkPEL-like* plants. We also calculated the NPQ, which was the same for both genotypes (**Figure 5B**). NPQ appeared quickly under short-term exposure to intense light and then relaxed in the same way in both genotypes.

### 3.6 Transcriptomic comparison of pQ35S::*TkPEL-like* and NIL plants

Given the clear negative impact of *TkPEL-like* overexpression on photosynthetic isoprenoid compounds in the leaf, but not on root isoprenoids, we investigated the underlying mechanism by comparing the transcriptomes of pQ35S::*TkPEL-like* and NIL leaves. We extracted RNA from the leaves of four individual 12-week-old plants from pQ35S::*TkPEL-like* lines #3.4, #4.1 and #5.7 and NILs as a control. The samples of each genotype were pooled so that two samples each representing 12 different plants were compared. The sequencing reads were mapped against the *T. koksaghyz* reference genome (Lin et al., 2022) with 93% efficiency, and were filtered for a minimum CPM of 0.2. The number of reads mapped to a gene was similar for both datasets (**Supplementary Figure S7**) and 30,093 transcripts could be detected for NIL plants and 29,830 for the pQ35S::*TkPEL-like* lines.

**Figure 6:**
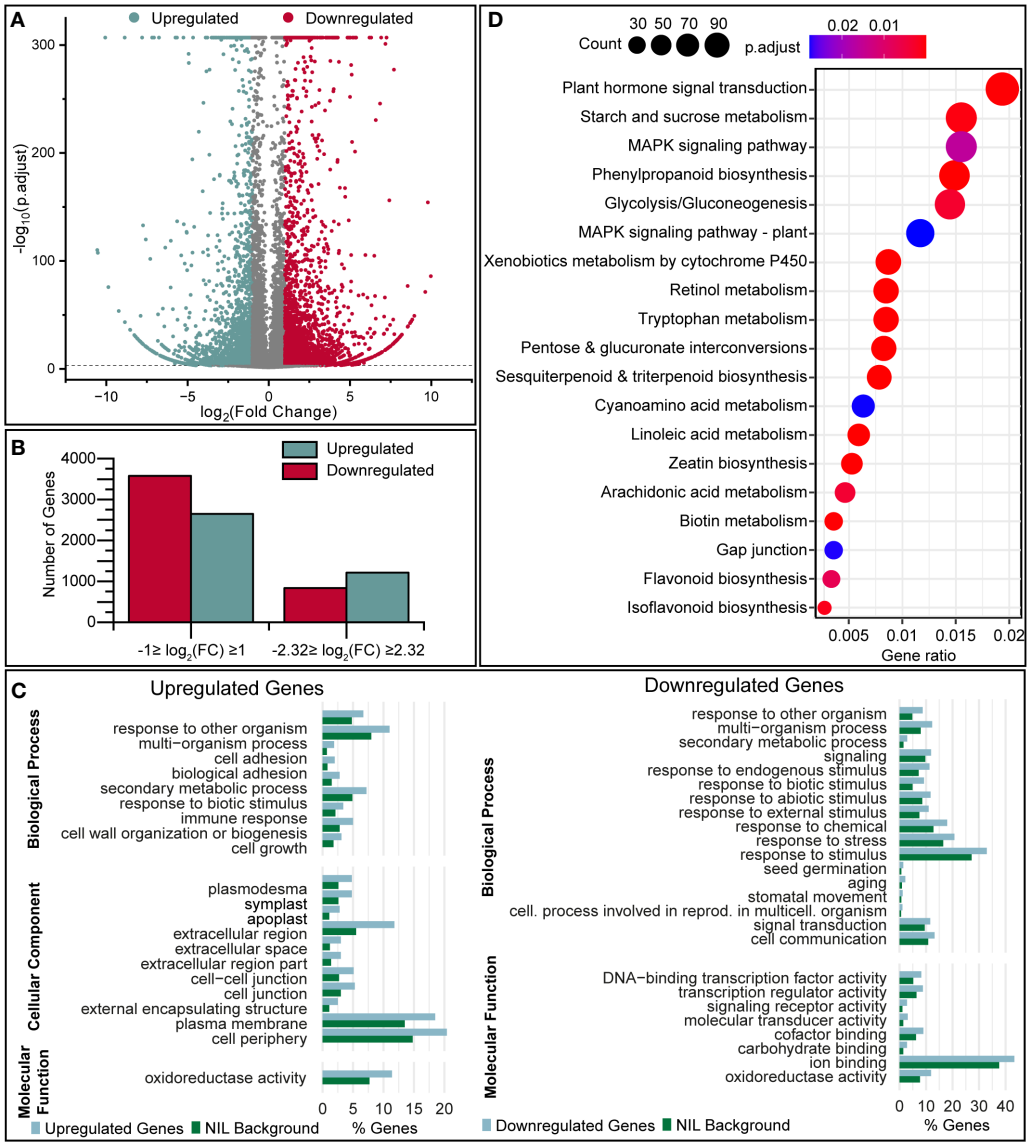
Transcriptomic analysis of pQ35S::*TkPEL-like* plants reveals a large number of differentially expressed genes. **(A)** Volcano plot showing the transcriptomic comparison of pQ35S::*TkPEL-like* and NIL plants. Log_2_(FC) values are plotted against –log_10_(p.adjust). Negative log_2_(FC) represents transcriptional upregulation in pQ35S::*TkPEL-like* plants compared to NIL. **(B)** Numbers of DEGs detected using different filtering criteria. Upregulation and downregulation refers to transcript levels in pQ35S::*TkPEL-like* plants. **(C)** GO terms enriched among DEGs (–5 ≥ FC ≥ 5) in pQ35S::*TkPEL-like* plants, showing terms of levels 1–3 significantly enriched (p < 0.05) among the DEGs compared to the NIL background. **(D)** KEGG pathways enriched among DEGs (–1 ≥ FC ≥ 1) in pQ35S::*TkPEL-like* plants. Gene ratios describe the number of genes assigned to a specific pathway compared to all genes that were assigned a pathway. Bubble size represents the absolute number of genes associated with the pathway and colors represent the adjusted p-value for enrichment compared to all transcripts identified in the NIL background. Pathways referring to processes in animals have been omitted.

Differentially expressed genes (DEGs) were defined as those meeting the threshold log_2_FC (normalized expression) ≥ 1 or ≤ –1 with a q-value (Storey) of < 0.05. We identified 2646 (upregulated) and 3580 (downregulated) genes that were modulated at least two-fold in the pQ35S::*TkPEL-like* plants vs NIL, of which 1215 (upregulated) and 837 (downregulated) genes were modulated at least five-fold (**Figures 6A, B**). For independent validation of the transcriptomic data, expression levels of single genes were analyzed in the three separate lines representing each genotype using qRT-PCR (**Supplementary Figure S8**). This confirmed the overall direction of transcriptional changes in pQ35S::*TkPEL-like* lines. Here it became obvious that, for specific genes, the transcriptional change seemed to be dependent on the level of *TkPEL-like* (over)expression.

Gene Ontology (GO) terms were compared with the background of all genes expressed in NIL leaves, showing that several terms were significantly (p < 0.05) enriched among the DEGs (**Figure 6C**). We focused on DEGs meeting the threshold –2.32 ≤ log_2_(FC) ≥ 2.32. Among the genes downregulated at least five-fold, we observed enriched GO terms in the superordinate category ‘Biological Process’ related to ‘signal transduction’, ‘signaling’ and ‘secondary metabolic process’ and responses to different stimuli such as ‘response to stress’, ‘response to abiotic/biotic stimuli’ and ‘response to endogenous/external stimuli’. Furthermore, the terms ‘response to other organism’, ‘multi-organism process’, ‘secondary metabolic process’, and ‘response to biotic stimulus’ were enriched for both the upregulated and downregulated genes, and the terms ‘cell growth’, ‘cell wall organization or biogenesis’ and terms related to ‘cell or biological adhesion’ were overrepresented only among the upregulated genes. Also among the downregulated genes, we observed enriched GO terms in the superordinate category ‘Molecular Function’ relating to ‘DNA-binding transcription factor activity’, ‘transcription regulator activity’ and ‘signaling receptor activity’, whereas the term ‘oxidoreductase activity’ was enriched for both the upregulated and downregulated genes. In the superordinate category ‘Cellular Component’, the upregulated genes were enriched for the terms ‘apoplast’, ‘cell junction’, ‘external encapsulating structure’ and ‘cell periphery’.

We also screened for KEGG pathways significantly enriched (p < 0.05) among the DEGs (–1 ≤ log_2_(FC) ≥ 1) compared to all genes expressed in the NIL plants (**Figure 6D**). We identified 42 pathways that were overrepresented among the DEGs, many of which related to processes and diseases only found in animals. This probably reflects the conservation of fundamental enzymes among eukaryotes, and such pathways were ignored for subsequent analysis. Enriched pathways present in plants included plant hormone signal transduction and the biosynthesis of phenylpropanoids, sesquiterpenoids, triterpenoids, flavonoids and isoflavonoids.

#### 3.6.1 Genes representing chlorophyll and carotenoid biosynthesis and related pathways are modulated in pQ35S::*TkPEL-like* leaves

**Figure 7:**
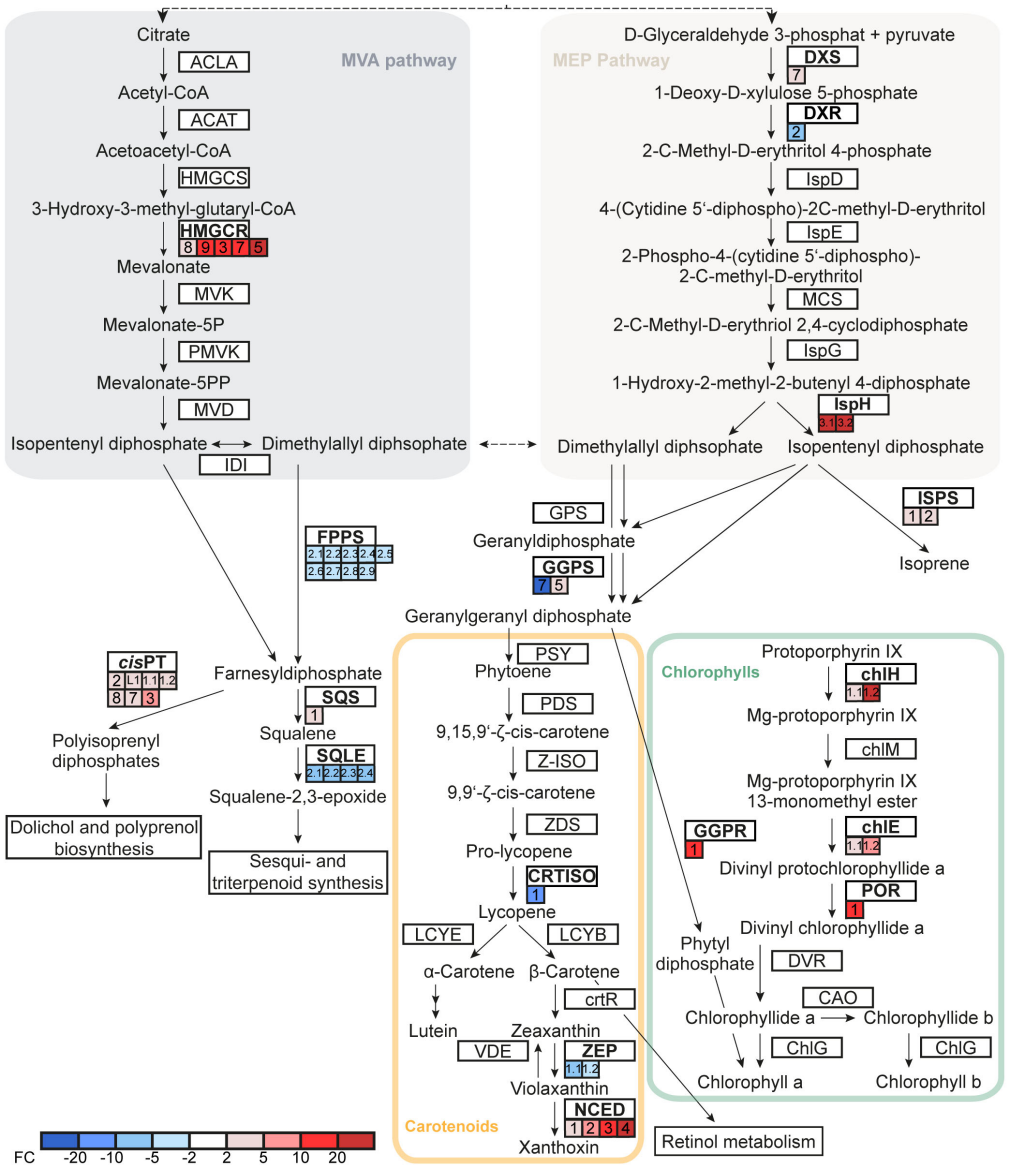
*TkPEL-like* overexpression affects genes involved in chlorophyll and carotenoid biosynthesis as well as precursors and connected pathways. Expression fold-changes of NIL compared to pQ35S::*TkPEL-like* plants are represented by colored boxes. Multiple boxes for one gene represent independent RNA IDs. Numbers in boxes assign transcripts to IDs based on the published genome (Lin et al., 2018) and **Supplemental Table S9**. ACLA, ATP citrate (pro-S)-lyase; ACAT, acetyl-CoA C-acetyltransferase; HMGCS, hydroxymethylglutaryl-CoA synthase; HMGCR, hydroxymethylglutaryl-CoA reductase; MVK, mevalonate kinase; PMVK, phosphomevalonate kinase; MVD, diphosphomevalonate decarboxylase; IDI, isopentenyldiphosphate δ-isomerase; DXS, 1-deoxy-d-xylulose-5-phosphate synthase; DXR, 1-deoxy-d-xylulose-5-phosphate reductoisomerase; IspD, 2-C-methyl-d-erythritol 4-phosphate cytidylyltransferase; IspE, 4-diphosphocytidyl-2-C-methyl-d-erythritol kinase; IspF, 2-C-methyl-d-erythritol 2,4-cyclodiphosphate synthase; IspG, (E)-4-hydroxy-3-methylbut-2-enyl-diphosphate synthase; IspH, 4-hydroxy-3-methylbut-2-en-1-yl diphosphate reductase; ISPS, isoprene synthase; GPS, geranyldiphosphate synthase; FPPS, farnesyldiphosphate synthase; GGPS, geranylgeranyldiphosphate synthase; PSY, phytoene synthase; PDS, 15-*cis*-phytoene desaturase; Z-ISO, ζ-carotene isomerase; ZDS, ζ-carotene desaturase; CRTISO, prolycopene isomerase; LCYB, lycopene β-cyclase; LCYE, lycopene ϵ-cyclase; crtR, β-carotene hydroxylase; ZEP, zeaxanthin epoxidase; NCED, 9-*cis*-epoxycarotenoid dioxygenase; VDE, violaxanthin de-epoxidase; chlH, Mg chelatase subunit H; chlM, Mg-protoporphyrin O-methyltransferase; chlE, Mg-protoporphyrin IX monomethyl ester cyclase; POR, protochlorophyllide reductase; GGPR, geranylgeranyldiphosphate reductase; DVR, divinyl chlorophyllide a 8-vinyl-reductase; CAO, chlorophyllide a oxygenase; ChlG, chlorophyll synthase; SQS, squalene synthase; SQLE, squalene monooxygenase; *cis*PT, *cis*-prenyltransferase.

The lower abundance of chlorophylls and carotenoids in pQ35S::*TkPEL-like* leaves suggests transcriptional changes in the MVA and MEP pathways as well as chlorophyll and carotenoid biosynthesis. In the MEP pathway, the genes representing the first step (*DXS*, encoding 1-deoxy-d-xylulose-5-phosphate synthase) and final step (*IspH*, encoding 4-hydroxy-3-methylbut-2-en-1-yl diphosphate reductase) were downregulated in the leaves of pQ35S::*TkPEL-like* plants, with *DXS* expression levels reduced by ~50% compared to the NIL control (**Figure 7**). In contrast, the gene responsible for the second step (*DXR*, encoding 1-deoxy-d-xylulose 5-phosphate reductoisomerase) was upregulated in these plants. On the other hand, *HMGR*, encoding hydroxymethylglutaryl-CoA reductase, the rate-limiting enzyme of the MVA pathway (Gondet et al., 1992; Suzuki et al., 2004; Hemmerlin et al., 2012), was also strongly repressed. Our data suggest that lower amounts of C_5_ isoprene diphosphate precursors are produced by the MEP and MVA pathways in leaves overexpressing *TkPEL-like*.

We detected four DEGs representing the downstream metabolic pathway leading to carotenoids. Interestingly, genes representing two different isoforms of geranylgeranyl diphosphate synthase (GGPS), which condenses four C_5_ isoprenoid precursors to geranylgeranyl diphosphate (GGPP) as a precursor for carotenoid biosynthesis, were antagonistically modulated in the pQ35S::*TkPEL-like* lines. Furthermore, the genes encoding prolycopene isomerase (*CRTISO*) and zeaxanthin epoxidase (*ZEP*) in the carotenoid pathway were strongly upregulated (**Figure 7**), whereas the *NCED* gene encoding 9-*cis*-epoxycarotenoid dioxygenase, which directs metabolic flux from the carotenoid pathway toward abscisic acid biosynthesis, was strongly repressed.

GGPP is not only a precursor for carotenoid biosynthesis but also for the production of intact chlorophyll *a* and *b*. It is reduced to phytyl diphosphate by geranylgeranyl diphosphate reductase (GGPR) and attached to chlorophyllide derived from protoporphyrin by chlorophyll synthase (Rüdiger, 1993; Keller et al., 1998). We found that *GGPR* and three genes involved in porphyrin ring synthesis were downregulated in leaves of pQ35S::*TkPEL-like* plants (**Figure 7**), providing a possible explanation for the lower chlorophyll levels (**Figure 4**). Genes representing competing metabolic pathways that build on farnesyl diphosphate (FPP, C_15_) as a substrate were also affected. For example, significant transcriptional changes were observed for the genes encoding squalene synthase and squalene monooxygenase, which direct metabolic flux towards triterpenoid and phytosterol synthesis, and *cis*-prenyltransferases, which form polyisoprenyl diphosphates essential for *N*-glycan synthesis and photosynthetic performance by modulating thylakoid membrane dynamics (Surmacz and Swiezewska, 2011; Akhtar et al., 2017; van Gelder et al., 2018).

#### 3.6.2 Several genes related to photosynthetic complexes, circadian rhythm and light regulation are downregulated in pQ35S::*TkPEL-like* leaves

Given the downregulation of photosynthesis-related genes in *Arabidopsis AtRPGE2* overexpression lines (Kim et al., 2023), the presence of a light-sensitive transcription factor binding site in the *TkPEL-like* promoter and the identification of several light-responsive genes among the DEGs involved in carotenoid and chlorophyll synthesis, we next focused on the analysis of genes involved in photosynthesis (other than chlorophyll biosynthesis), circadian rhythm and light-dependent signaling (**Supplemental Table S6**). Notably, we observed the downregulation of the genes *COP1* and *PIF1*, which as discussed above encode negative regulators of photomorphogenesis. Regulators of the circadian clock, which control flowering time, were also strongly downregulated in the *TkPEL-like* overexpression lines, including *ELF3*, *CO* and *FT*. Several FAR1-related sequence (*FRS*) homologs, encoding proteins that interact with AtRPGE2 (Dreze et al., 2011), were also downregulated. Interestingly, although GLK1 and GLK2 interact with AtRPGE2 and are transcriptionally correlated with *AtRPGE2* overexpression (Kim et al., 2023), we did not detect this sequence among the DEGs. However, we found that a number of photosynthesis-related genes, including those encoding subunits of PS I and II, light-harvesting complex II chlorophyll *a*/*b* binding protein 1, and ATPase subunits, were repressed. Similar profiles were reported for the *AtRPGE2* overexpression lines (Kim et al., 2023). Only genes encoding a cytochrome b6-f complex iron-sulfur subunit and a photosystem I subunit III were induced by *TkPEL-like* overexpression (**Supplemental Table S7**). Other DEGs encoded homologs of transcription factor MYB75/PAP1 (which may be transcriptionally regulated by HY5). MYB75/PAP1 is a master regulator of anthocyanin biosynthesis and targets *CHS* among other genes (Shin et al., 2013).

#### 3.6.3 RNA degradation genes are differentially expressed in pQ35S::*TkPEL-like* leaves

Initial homology searches revealed similarities between TkPEL-like and a polyribonucleotide nucleotidyltransferase, also known as polyribonucleotide phosphorylase (PNPase), which is involved in RNA maturation and degradation (Bollenbach et al., 2004; Germain et al., 2011). Therefore, we screened genes representing the RNA degradation and surveillance pathways to see if they were also modulated by *TkPEL-like* overexpression (**Supplemental Table S8**). Enolase and 6-phosphofructokinase are thought to form an RNA degradosome complex with PNPase and other proteins in *Bacillus subtilis*, and *T. koksaghyz* homologs were found to be differentially expressed in pQ35S::*TkPEL-like* plants. In addition, the DEGs included several cofactors of RNA exosomes and subunits of the Ccr4-NOT complex, an important modulator of gene expression at the mRNA level (Collart, 2016). One particular downregulated gene encoded a putative ATP-dependent RNA helicase DOB1/MTR4 homolog. In yeast, the helicase activity of this protein and its ability to act as a cofactor in poly(A)polymerase complexes involved in RNA degradation are thought to be important for the function of exosomes (Vaňáčová et al., 2005; Falk et al., 2017; Schmid and Jensen, 2019). In addition, a poly(A)-binding protein with multiple functions in gene regulation (e.g., by mediating poly(A) tail synthesis and nuclear mRNA export) was upregulated in the pQ35S::*TkPEL-like* plants (Mangus et al., 2003).

## 4 Discussion

### 4.1 The *TkPEL-like* overexpression phenotype resembles the effects of *AtRPGE2* and *DCAR_032551* in other plants

Recent progress has provided insight into the complexity of light-dependent regulation and isoprenoid biosynthesis in plants (reviewed by Vranová et al., 2012; Tholl, 2015; Li et al., 2022; Wang et al., 2022). Previous studies have indicated that the carrot protein DCAR_032551 (Iorizzo et al., 2016) and the *Arabidopsis* protein RPGE2 (Ichikawa et al., 2006; Kim K. et al., 2016; Kim et al., 2023) are involved in light signal transduction and the regulation of isoprenoids and isoprenoid-containing compounds such as carotenoids and chlorophylls. We have now identified TkPEL-like, a putative homolog of DCAR_032551/AtRPGE2 in *T. koksaghyz*. The overexpression of *TkPEL-like* in *N. benthamiana* and *T. koksaghyz* led to a pale green leaf phenotype and a reduction in chlorophyll and carotenoid levels (**Figure 4**). This indicates the cross-species functional conservation of this protein in photosynthetic organisms, which is also supported by the expression of rice (*Oryza sativa*) *RPGE* in *Arabidopsis* and the interaction between RPGE and GLK homologs of different species (Kim et al., 2023). However, the faster stem elongation and early flowering observed in *Arabidopsis* plants overexpressing *AtRPGE2* (Ichikawa et al., 2006) was not observed in our pQ35S::*TkPEL-like T. koksaghyz* plants. Contrary to the normal flowering time, different genes involved in the circadian rhythm, which regulate flowering in other plants, were among the DEGs in pQ35S::*TkPEL-like* vs. NILs (**Supplementary Table S7**). To our knowledge, the control of flowering time in *T. koksaghyz* is not yet understood and we do not know the exact functions of the corresponding genes because different functions have been described for different isoforms (Beinecke et al., 2018; Lin et al., 2019; Schmidt et al., 2020). The vernalization-dependent flowering of *T. koksaghyz* appears to play an important role, but the various protein isoforms that regulate flower development in *T. koksaghyz* require further detailed analysis.

*DCAR_032551* is more strongly expressed in highly pigmented carrot roots compared to pale roots, but the gene carries frameshift mutations that probably cause functional disruption (Iorizzo et al., 2016). The absence of a functional *DCAR_032551* gene product therefore appears to be associated with high carotenoid levels whereas a functional DCAR_032551 protein has the opposite effect. This matches the phenotype of *TkPEL-like* overexpression in *T. koksaghyz*. Furthermore, neither the carrot nor dandelion plants discussed above showed any developmental abnormalities, suggesting that the synthesis of photosynthetic isoprenoids is affected rather than photomorphogenesis as a whole. However, in contrast to carrots, the isoprenoid content of *T. koksaghyz* roots was unaffected by *TkPEL-like* overexpression (**Supplementary Figure S6**). The transcriptional downregulation of the MVA pathway (as detected in the leaves) may therefore be restricted to green tissues and root isoprenoid synthesis might be regulated differently. MEP pathway downstream products such as carotenoids have not been identified as typical latex/root metabolites, although some MEP pathway proteins have been detected in *T. koksaghyz* latex (Niephaus et al., 2019; Xie et al., 2019).

### 4.2 *TkPEL-like* overexpression may primarily repress chlorophyll and isoprenoid precursor biosynthesis resulting in the secondary induction of carotenoid biosynthesis genes

Transcriptomic analysis of pQ35S::*TkPEL-like* lines revealed the downregulation of genes involved in chlorophyll biosynthesis and the MEP and MVA pathways in leaves, providing an explanation for the observed phenotype. In highly pigmented carrots, the *DXS* gene (representing the first step in the MEP pathway) was induced, whereas in the pale green pQ35S::*TkPEL-like* dandelion plants it was strongly suppressed (Iorizzo et al., 2016). This suggests TkPEL-like is a negative regulator of *DXS*, and that other transcriptional changes may be secondary effects. For example, inhibiting the MEP pathway in *Arabidopsis* revealed that the MEP and tetrapyrrole pathways are co-regulated to maintain a metabolic balance and to prevent photo-oxidative damage by intermediates (Kim et al., 2013).

*IspH* expression was also strongly reduced in the pQ35S::*TkPEL-like* plants, in contrast to the outcome in carrots. In *Arabidopsis* and tomato (*Lycopersicon esculentum*), IspH is a key enzyme in MEP-derived plastidial isoprenoid biosynthesis, and the upregulation of *IspH* increased the incorporation of isoprenoid precursors into a downstream recombinant pathway by 100% compared to the upregulation of *DXS* (Estévez et al., 2001; Botella-Pavía et al., 2004; Kishimoto and Ohmiya, 2006; Banerjee and Sharkey, 2014). The simultaneous downregulation of *IspH* and *DXS* may have reduced the allocation of IPP and DMAPP from the MEP pathway, which could not be compensated by the upregulation of *DXR* due to the limited amount of substrate.

The downregulation of chlorophyll biosynthesis genes and other photosynthesis-related genes was similar to the transcriptional changes observed following *AtRPGE2* overexpression in *Arabidopsis*, although not all the genes downregulated in *Arabidopsis* were among the DEGs in *T. koksaghyz* (Kim K. et al., 2016; Kim et al., 2023) (**Supplementary Table S7**). In *Arabidopsis*, RPGE2 prevents GLK target gene activation by the formation of heterodimers, which probably contributed to the transcriptional changes observed following *AtRPGE2* overexpression (Kim et al., 2023). The upregulation of *GLK* as described in Arabidopsis could not be detected in our RNA-seq data. To test for a conserved molecular mode of action for TkPEL-like, as suggested in rice (Zhang et al., 2021), GLK homologs in *T. koksaghyz* and their potential interaction with TkPEL-like should be investigated in the future.

The higher maximum quantum yield in pQ35S::*TkPEL-like* plants compared to NIL controls (**Figure 5**) was likewise reported for plants overexpressing AtRPGE2 (Kim et al., 2023). However, the associated lower total seed yields reported for *Arabidopsis* were not evaluated in our experiments. The increase in F_v_/F_m_ indicates an increase of the proportion of the absorbed light energy that is used for photochemical reactions, which could be a consequence of the lower chlorophyll content in order to maintain sufficient photosynthesis. In chlorophyll-deficient rice mutants, an increased photosynthetic rate per chlorophyll molecule compensated for the negative impact of chlorophyll reduction (Li et al., 2013). It is possible that such an increase also contributed to sufficient photosynthesis and normal biomass accumulation in pQ35S::*TkPEL-like* plants (**Supplementary Figure S5**). The lower chlorophyll content could have also reduced the photochemical damage and heat stress in leaves absorbing more light than required for maximum photosynthesis, and energy otherwise used for repair could therefore be used elsewhere, contributing to the comparable biomass accumulation in pQ35S::*TkPEL-like* and NILs (Hamblin et al., 2014). The comparable NPQ in the pQ35S::*TkPEL-like* plants and NIL controls supports the hypothesis that *TkPEL-like* overexpression does not trigger stress. This is particularly notable given that many essential photosynthetic components, such as plastoquinone (an intramembrane electron acceptor downstream of PSII) are also derived from the MEP pathway that was transcriptionally downregulated in pQ35S::*TkPEL-like* plants (Nowicka and Kruk, 2010).

Despite the lower carotenoid levels in pQ35S::*TkPEL-like* plants, carotenoid pathway genes tended to be upregulated. This may also be a primary effect, but we hypothesized it might have been a secondary feedback mechanism in response to low carotenoid levels (Avendaño-Vázquez et al., 2014). However, the limited pool of isoprenoid precursors may not have allowed for compensation via transcriptional upregulation. The possible upregulation of *CCD*, encoding a carotenoid cleavage dioxygenase, suggests an exacerbation of low carotenoid synthesis, given that this enzyme is responsible for the degradation of carotenoids in chrysanthemum, resulting in white petals (Ohmiya et al., 2006).

Three DEGs were found in the pathway leading from IPP and DMAPP to the formation of the first carotenoids. IPP and DMAPP are condensed by prenyltransferases to form pools of C_10_ (geranyl diphosphate), C_15_ (FPP) and C_20_ (GGPP) precursors (Hemmerlin et al., 2012). For carotenoid biosynthesis, two GGPP molecules are condensed by phytoene synthase (PSY) to form phytoene, which is desaturated and isomerized in several steps (Shumskaya and Wurtzel, 2013). Interestingly, the genes encoding FFP synthase (*FDPS*) and GGPP synthase (*GGPS*) were upregulated in leaves of pQ35S::*TkPEL-like* plants, possibly to increase the flux from IPP/DMAPP in this direction. Another *GGPS* isoform was downregulated in the transgenic plants. Given the widespread occurrence of *GGPS* gene families together with the specialization of the individual enzymes (Beck et al., 2013; Ruiz-Sola et al., 2016), the opposing transcriptomic responses of the dandelion *GGPS* genes may reflect their spatial expression profiles or protein localization. In tobacco (*Nicotiana tabacum*), NtGGPPS1 and a light-regulated small subunit of GGPS were shown to interact with PSY, allowing the channeling of GGPP between successive pathway enzymes. The differential expression of *GGPPS* may therefore affect the efficiency of PSY in pQ35S::*TkPEL-like* plants, although the *PSY* gene itself was not differentially expressed. The specific functions of the differentially regulated isoforms should be examined in detail to validate the hypotheses derived from our expression data.

The induced genes in the downstream parts of the carotenoid pathway included *ZEP*, encoding zeaxanthin epoxidase (responsible for violaxanthin biosynthesis) whereas the gene encoding 9-cis-epoxycarotenoid dioxygenase (which converts violaxanthin to xanthoxin) was suppressed, indicating that violaxanthin levels were specifically increased in the pQ35S::*TkPEL-like* lines. In the xanthophyll cycle, violaxanthin is reversibly de-epoxidized to zeaxanthin by violaxanthin de-epoxidase under intense light to efficiently reduce photo-oxidative stress and lipid peroxidation (Havaux et al., 2007). The induction of *ZEP* may therefore prepare the cell to deal with reactive oxygen species by providing enough substrate for the detoxifying reaction that converts violaxanthin to zeaxanthin, and the limited quantity of carotenoids available was optimally deployed.

Finally, genes involved in flavonoid/isoflavonoid metabolism were also significantly upregulated in pQ35S::*TkPEL-like* plants compared to NIL controls (**Figure 6B**). One example is the key enzyme CHS, which also plays a role in cell wall organization and biosynthesis (Zuk et al., 2016), a process significantly enriched among upregulated genes (**Figure 6C**). *CHS* is also regulated by light, via HY5 and COP1 (Ang et al., 1998; Thain et al., 2002). These data suggest that flavonoid pigments, such as anthocyanins, were enriched to compensate for the low carotenoid levels in the plastids thus ensuring photoprotection, given there was no difference in fitness between the pQ35S::*TkPEL-like* plants and NILs. Such compensatory functions have been proposed in response to UV radiation (Guidi et al., 2016).

### 4.3 TkPEL-like may act downstream of HY5 to enable light-dependent regulation

*TkPEL-like* overexpression was negatively correlated with the expression of various modulators of the light response, in contrast to the highly pigmented carrots in which there was a positive correlation (Iorizzo et al., 2016). Transcription factors (HY5 and PIFs) and the E3 ubiquitin ligase COP1 are key regulators of the light response. PIFs negatively regulate photomorphogenesis and repress the MEP pathway (Chenge-Espinosa et al., 2018), as well as chlorophyll and carotenoid biosynthesis (Huq et al., 2004; Moon et al., 2008; Toledo-Ortiz et al., 2010; Tang et al., 2012; Job and Datta, 2021). PIF1 and PIF3 repress transcription, and their degradation in the presence of light thus enables the expression of MEP pathway genes such as *IspH* (Chenge-Espinosa et al., 2018). In *pif1* and *pif3* mutant seedlings, protochlorophyllide accumulates in the dark, leading to bleaching under illumination, in parallel to higher levels of light-induced chlorophyll and increased carotenoid accumulation in the dark (Huq et al., 2004; Toledo-Ortiz et al., 2010; Job and Datta, 2021).

PIFs are also potential COP1 cofactors that represses photomorphogenesis in the dark by accumulating in the nucleus, facilitating the degradation of positive regulators of light signaling such as HY5 (Deng et al., 1991; Osterlund et al., 1999; Saijo et al., 2003; Jang et al., 2005; Xu et al., 2014). Accordingly, *cop1* mutants show photomorphogenesis in the dark (Deng et al., 1991). These reports contrast with the indicated downregulation of *PIF* and *COP1* in pale green pQ35S::*TkPEL-like* plants. The identification of a G-box element in the *TkPEL-like* promoter hints that *TkPEL-like* may be regulated by HY5, PIFs, or both. *HY5* expression was unaffected in pQ35S::*TkPEL-like* plants, suggesting that HY5 may act upstream of TkPEL-like in the regulatory hierarchy. In contrast, *PIFs* were differentially expressed in the pQ35S::*TkPEL-like* plants, but *AtRPGE2* was identified as a direct target of PIFs (Kim K. et al., 2016). The exact relationship between PIFs and *TkPEL-like* should be analyzed in future studies to gain a more detailed understanding of this regulatory network in *T. koksaghyz*.

One explanation for these contradictory results is that *COP1* and *PIF1* were downregulated as a feedback response to low carotenoid and chlorophyll levels in order to de-repress photomorphogenesis and associated pigment synthesis, but the limited pool of isoprenoid precursors prevented the normalization of carotenoid and chlorophyll levels. Furthermore, although three transcripts annotated as *PIF1* or *PIF3* with sequence identities > 50% were indeed downregulated in pQ35S::*TkPEL-like* plants, one further transcript with high similarity to the ATP-dependent DNA helicase PIF1-like from *Cynara cardunculus* var. *scolymu*s was strongly upregulated. The overexpression of a single *PIF1* isoform together with *TkPEL-like* may have been sufficient to effectively repress photomorphogenesis, including the downregulation of MEP/MVA and chlorophyll biosynthesis pathway gene expression. However, we found no evidence for the transcriptional modulation of PIF target genes such as *PSY*, as described in other plants (Toledo-Ortiz et al., 2010). *PSY*, among other photomorphogenic targets, was shown to be antagonistically regulated by PIFs and HY5 (Toledo-Ortiz et al., 2014). Therefore, unaffected *HY5* expression may have been sufficient to regulate HY5 targets in a normal manner, despite differential *PIF* expression. This would further support the idea that TkPEL-like acts downstream of HY5 in the signaling hierarchy.

We observed the downregulation of *COP1*, but this gene was co-expressed with nonfunctional *DCAR_032551* in highly pigmented carrots (Iorizzo et al., 2016). COP1 is excluded from the nucleus under prolonged illumination (Osterlund and Deng, 1998; Subramanian et al., 2004), so the transcriptional modulation of *COP1* in response to light may not be critical for photomorphogenesis as long as nuclear exclusion is not impaired. Notably, translational and post-translational regulation can also influence metabolic pathways in a way that is not fully evident from our data (Hemmerlin et al., 2012; Shumskaya and Wurtzel, 2013; Banerjee and Sharkey, 2014).

### 4.4 TkPEL-like may play a role in RNA degradation

TkPEL-like is related to a PNPase and the presence of a PIN_Fcf1-like domain prompted us to analyze genes involved in mRNA processing. This indeed revealed several DEGs that are homologs of genes involved in RNA degradation and surveillance. PNPases play various roles in RNA metabolism. In *Arabidopsis*, PNPase was shown to facilitate plastid mRNA, tRNA and 23S rRNA metabolism, including poly(A) tail formation and degradation, as well as RNA degradation in general (Walter et al., 2002; Holec et al., 2006; Germain et al., 2011). Knockout mutants revealed a phenotype similar to pQ35S::*TkPEL-like* plants, with pale young leaves but near normal mature tissues (Sauret-Güeto et al., 2006). The PIN_Fcf1-like domain (~120 residues) may facilitate pre-rRNA cleavage, nonsense mediated mRNA decay and RNA interference (Clissold and Ponting, 2000; Fatica et al., 2004). However, the level of similarity between TkPEL-like, and PNPase and the PIN_Fcf1-like domain was only low, and PNPases are much larger (e.g., 922 amino acids for AtPNPase) than the 108-amino-acid TkPEL-like protein (Walter et al., 2002). Furthermore, plant PNPases have only been found in chloroplasts and mitochondria (Li et al., 1998; Yehudai-Resheff et al., 2001; Holec et al., 2006; Germain et al., 2012), whereas our data suggest that TkPEL-like is localized in the nucleus and cytosol. The secondary structure of the PIN_Fcf1-like domain also differs from that predicted for TkPEL-like (**Figure 1**) (Senissar et al., 2017). However, a number of conserved acidic residues are found in both sequences. These data suggests that the function of TkPEL-like differs from that described in other species.

### 4.5 TkPEL-like probably suppresses genes involved in chlorophyll and isoprenoid precursor biosynthesis through its conserved N-terminal domain in a light-dependent manner

Our results support the hypothesis that the protein family containing AtRPGE2 and TkPEL-like acts as a negative regulator of photomorphogenesis (specifically chlorophyll and carotenoid accumulation) and represents an early step in the light-dependent signal transduction system (Iorizzo et al., 2016). The involvement of TkPEL-like proteins in light signal transduction and photomorphogenesis would also explain why this protein family is almost exclusive to photosynthetic organisms thus far, with highly conserved functions (Kim et al., 2023). However, RNAi-induced knockdown in *Arabidopsis* (Ichikawa et al., 2006) and our attempt to generate *T. koksaghyz* knockdown lines consistently resulted in a lethal phenotype, whereas an *Atrpge1/2/3* triple mutant was viable (Kim et al., 2023). The phenotype of the triple mutant contrasted with the results of overexpressing *AtRPGE1*, *AtRPGE2* or *TkPEL-like*, which suggested partial redundancy among the different isoforms in *Arabidopsis* (Kim et al., 2023). Additionally, highly pigmented carrots expressing a presumably nonfunctional version of *DCAR_032551* were also viable (Iorizzo et al., 2016). This might reflect a functional specialization of DCAR_032551 so that only root isoprenoids derived from the MEP pathway are affected. The authors did not report low chlorophyll levels in the aboveground organs, and *TkPEL-like* overexpression did not influence MVA pathway isoprenoids in *T. koksaghyz* roots.

Predominant expression of *TkPEL-like* in leaves from the ninth week onwards (**Figure 5B**) coincided with the development of a visible leaf rosette under our greenhouse conditions (**Supplementary Figure S5**). This expression profile correlated with that of *AtRPGE2* (Klepikova et al., 2016). Given the hypothesis that the corresponding protein is a repressor of photosynthetic gene expression, de-etiolation and photomorphogenesis, low expression levels in young plants may promote leaf development and coloration to cope with the initial exposure to light, and higher expression levels in mature leaves are necessary to restrict the aforementioned processes to an appropriate level.

Our data also support recent findings on the molecular function of AtRPGE2 (Kim et al., 2023). The interaction between AtRPGE2 and GLK in the cytosol and nucleus of *N. benthamiana* cells agrees with our results showing the localization of Cerulean–TkPEL-like fusion proteins in the cytosol and the nucleus in the same species. However, TkPEL-like lacks a nuclear localization signal, and may be transported into the nucleus passively due to its small size or actively transported in response to light, as reported for photoreceptors (Genoud et al., 2008; Galvão and Fankhauser, 2015).

TkPEL-like mutants with either one or two conserved cysteines replaced did not suppress chlorophyll or carotenoid biosynthesis to the same degree as the wild-type protein, suggesting the cysteine residues are functionally important (**Figure 3**). The cysteine residues found in the N-terminal segment of TkPEL-like are highly conserved between *Arabidopsis*, carrot and *T. koksaghyz* (**Figure 1A, B**), and may therefore be required for their molecular function. The conserved cysteine residues could also serve as points of attack for redox signaling (Couturier et al., 2013), which is known to regulate transcription, posttranslational modifications and retrograde signaling (Surpin et al, 2002; Balmer et al., 2003; Lemaire et al., 2004).

### 4.6 Conclusions

TkPEL-like is a promising candidate for the regulation of isoprenoid biosynthesis in leaves because it affects leaf isoprenoid but not root isoprenoid levels when constitutively overexpressed. It may act within the light-dependent signal transduction pathway and react to the redox status of the cell, thereby enabling responses to environmental cues. Our transcriptomic data provide a broad overview of the pathways affected by *TkPEL-like* overexpression and can be used as a basis for future functional studies to validate our hypotheses and to fully understand the complex regulatory network controlling isoprenoid biosynthesis and photomorphogenesis. A better understanding of the metabolic network will facilitate future breeding approaches aiming to modulate plant isoprenoid levels such as chlorophylls and carotenoids, or possibly other metabolites that we have not studied yet. It could also lead to the development of a suitable plant-based production platform for valuable metabolites.

## Data availability statement

The original contributions presented in the study are publicly available. This data can be found here: https://www.ncbi.nlm.nih.gov/sra/PRJNA985648.

## Author contributions

SMW, VAB, K-UR and CSG conceived and designed the experiments. SMW, VAB and NvD conducted the experiments. SMW, VAB and K-UR analyzed the data. NvD, DP and CSG contributed the reagents, materials, and analytical tools. SMW and RMT wrote the manuscript. All authors contributed to the article and approved the submitted version.
