# Supplementary material for: Overexpression of a pseudo-etiolated-in-light-like protein in Taraxacum koksaghyz leads to a pale green phenotype and enables transcriptome-based network analysis of photomorphogenesis and isoprenoid biosynthesis
Source: Front Plant Sci. 2023 Sep 28;14:1228961. doi: 10.3389/fpls.2023.1228961 (PMC10569127; doi:10.3389/fpls.2023.1228961)
Supplement: Supplementary file 1 [file DataSheet_1.docx]

Supplementary Material

**Overexpression of a pseudo-etiolated-in-light-like protein in *Taraxacum koksaghyz* leads to a pale green phenotype and enables transcriptome-based network analysis of photomorphogenesis and isoprenoid biosynthesis**

Wolters, S.M.^1a^, Benninghaus, V.A.^1a^, Roelfs, K.U.^1^, van Deenen, N.^2^, Twyman R.M.^3^, Prüfer, D.^1,2^, Schulze Gronover, C.^1*^

^1^Fraunhofer Institute for Molecular Biology and Applied Ecology IME, Münster, Germany

^2^Institute for Biology and Biotechnology of Plants, University of Münster, Münster, Germany

^3^ TRM Ltd, Scarborough, United Kingdom

^a^These authors contributed equally to the article

*** Correspondence:**Corresponding Author
christian.schulze.gronover@ime.fraunhofer.de

# Supplementary Data

Supplementary Data S1 can be found in a separate Excel file.

# Supplementary Figures and Tables

## Supplementary Figures


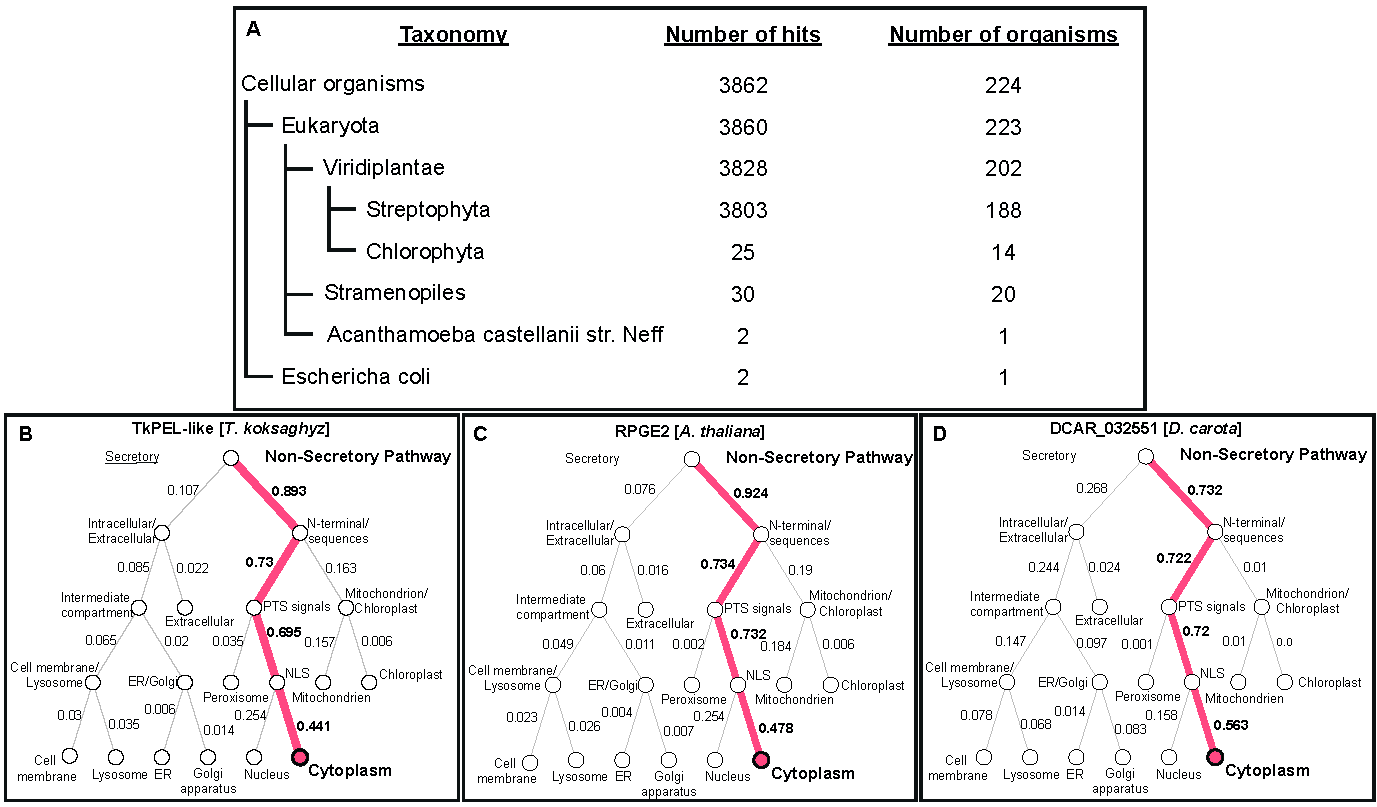


Supplementary Figure S1. *In silico* analysis of TkPEL-like. (A) Protein sequence similarity search of TkPEL-like against the NCBI nr database. The phylogenetic tree visualizes the BLAST hit taxonomy. Max target sequences = 20,000, E-threshold = 10, word size = 6, matrix = BLOSUM62, E-value cut off = 0.001. (B, C, D) Subcellular localization prediction for TkPEL-like, AtRPGE2 and DCAR_032551 performed by DeepLoc.


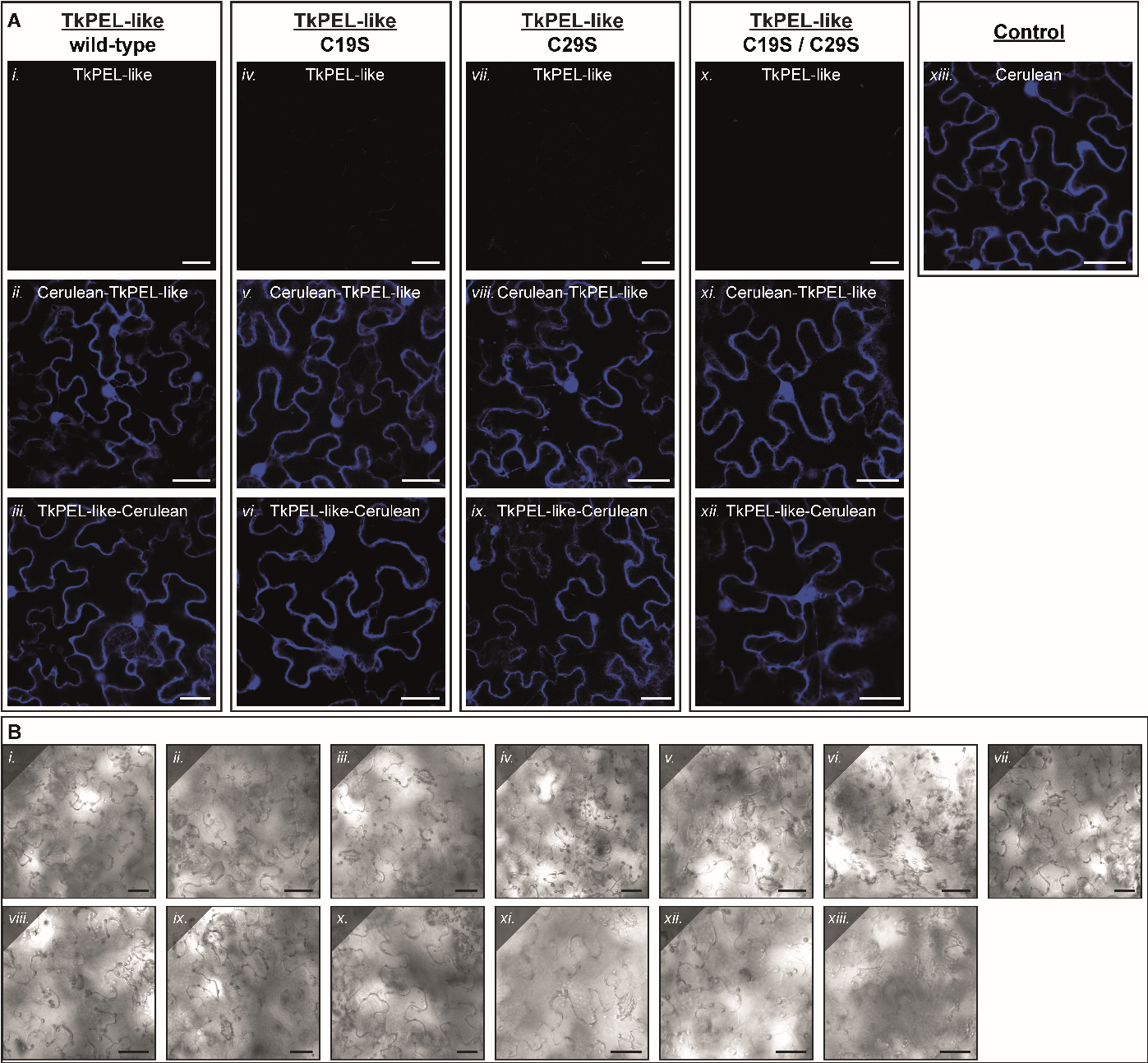


Supplementary Figure S2. Heterologous expression of *TkPEL-like* mutants in *N. benthamiana* leaf epidermis cells results in cytosolic and nuclear protein localization. (A) Confocal laser scanning microscopy images of leaves expressing N-terminal and C-terminal TkPEL-like Cerulean fusions of the wild-type and mutant sequences and a Cerulean control. (B) Bright-field images corresponding to confocal laser scanning microscopy images in A. *i.-xiii.* refer to labels in A. Scale bar = 40 μm.


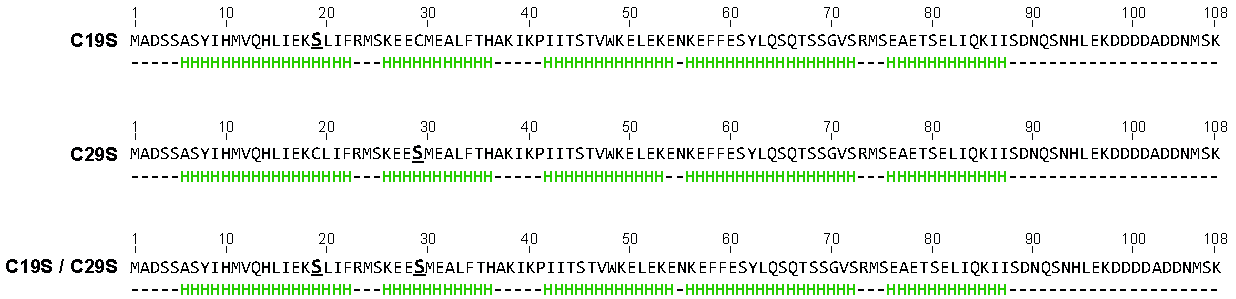


Supplementary Figure S3. Secondary protein structure prediction of cysteine-mutated TkPEL-like proteins. The cysteine to serine substitutions are underlined and highlighted in bold. The predicted α-helices are shown in green (H). The prediction was performed by JPred 4.


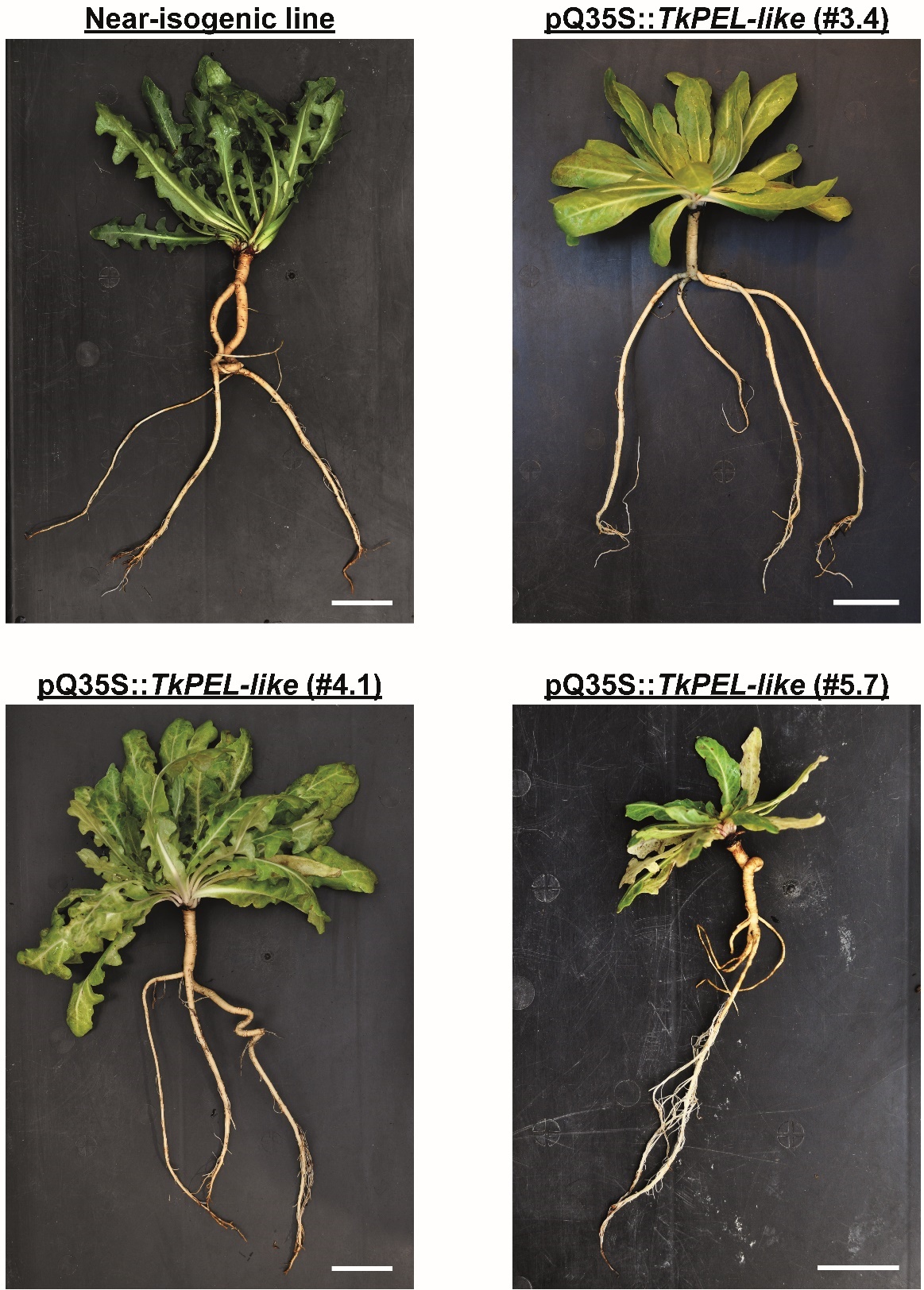


Supplementary Figure S4. Plant morphology of three 12-week-old pQ35S::*TkPEL-like* *T. koksaghyz* and one NIL control of the T_2_ generation. Plants were grown under controlled greenhouse conditions. Scale bar = 3.6 cm.


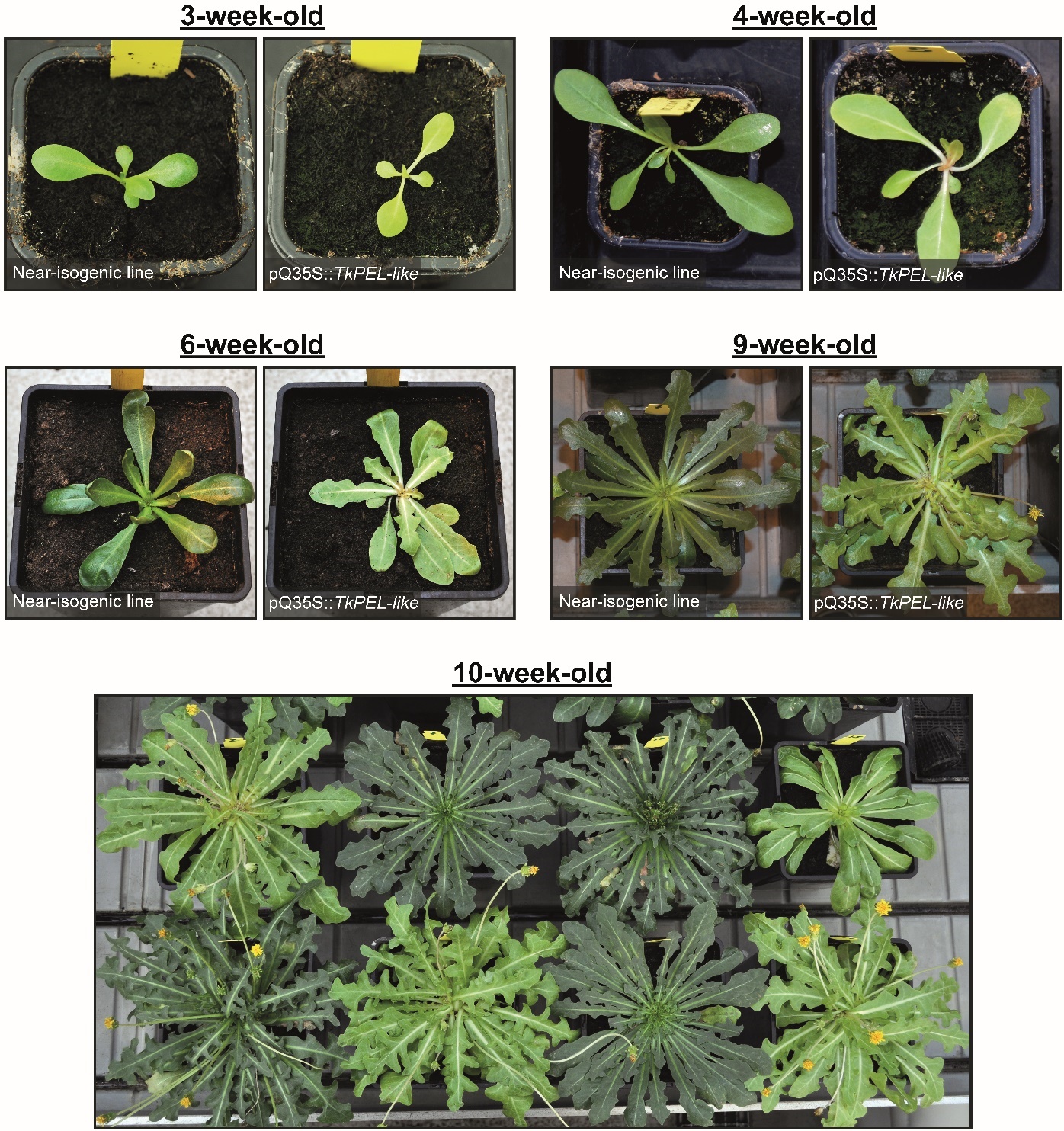


Supplementary Figure S5. Leaf morphogenesis of pQ35S::*TkPEL-like* *T. koksaghyz* (#3.4) and NIL controls of the T_2_ generation. Plants were cultivated under controlled greenhouse conditions. The leaf morphology was monitored after 3, 4, 6, 9 and 10 weeks.


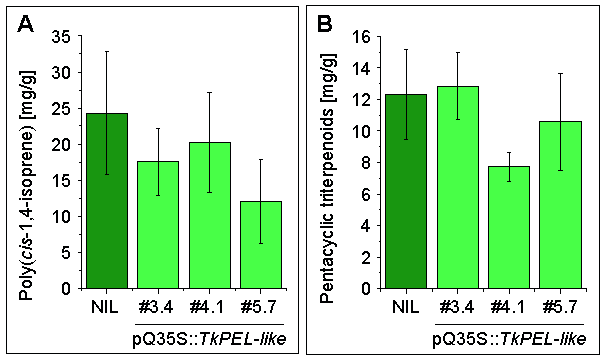


**Supplementary Figure S6.** Secondary metabolite concentrations in roots of pQ35S::*TkPEL-like* *T. koksaghyz* do not differ from NIL controls. For pQ35S::*TkPEL-like* lines #3.4, #4.1 and #5.7 samples of two individual plants each were pooled and analyzed in four independent extractions. For NIL controls ten individual plants were analyzed. Data are means (±SD). **(A)** Poly(*cis*-1,4-isoprene) concentrations. **(B)** Pentacyclic triterpenoid concentrations calculated based on the detection of α- and β-amyrin, lupeol, lup(19,21)-en-3-ol, taraxasterol, taraxerol and further unidentified triterpenoids (Supplementary Table S6). Statistical differences were assessed by non-parametric Mann-Whitney U-tests and a significance level of **P < 0.01.


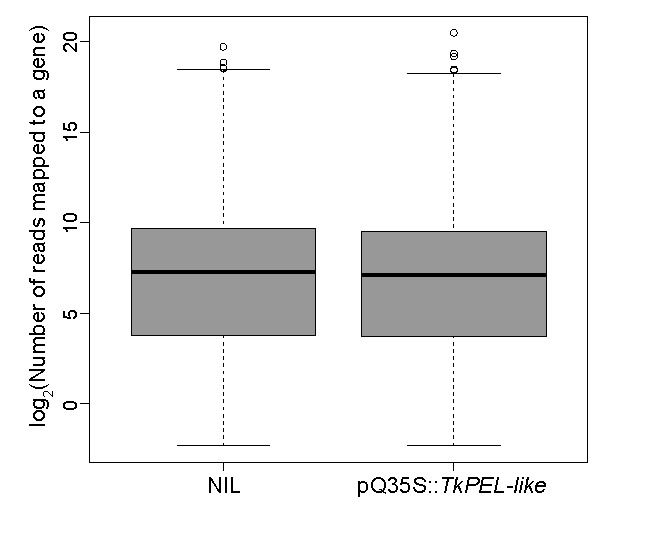


Supplementary Figure S7. Number of reads mapped to a gene for pQ35S::*TkPEL-like* and NIL data.


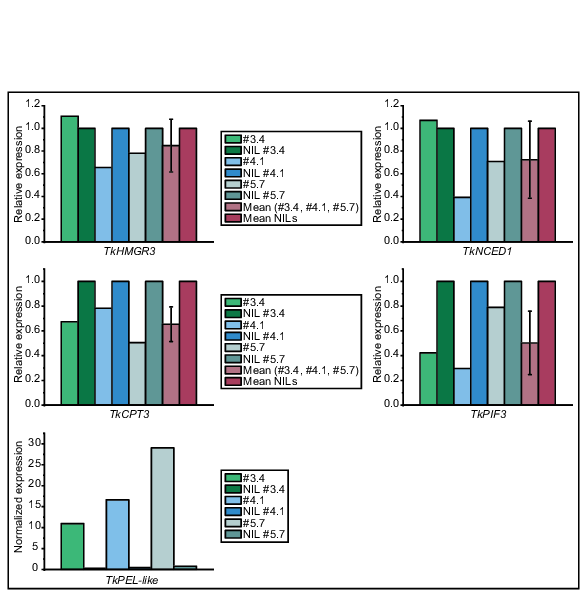


Supplementary Figure S8. Validation of transcriptomic data by qRT-PCR. Relative expression of a set of DEGs in pQ35S::*TkPEL-like* lines compared to NILs. Mean (#3.4, #4.1, #5.7) represents the mean transcriptional change in the different transgenic lines compared to the respective NIL. In parallel, *TkPEL-like* (over)expression was quantified. Three single pools of leaf RNA of each genotype (pQ35S::*TkPEL-like* and NIL) were used for the analysis. Expression levels were normalized against *elongation factor 1a* (*TkEf1a*) and *ribosomal protein L27* (*TkRP*).

## Supplementary Tables

Supplementary Table S1. Oligonucleotides used in this study.

Supplementary Table S2. Hits of BLAST searches against the NCBI nr/nt databases.

| Description | Organism | Query Coverage | E-value | % Identity | Accession No. |
| --- | --- | --- | --- | --- | --- |
| Protein BLAST | | | | | |
| DNA replication complex GINS protein [*Salix suchowensis*] | *Salix suchowensis* | 93% | 4x10^-40^ | 70.30% | KAG5237648.1 |
| putative angiotensin-converting enzyme 2 [*Helianthus annuus*] | *Helianthus annuus* | 91% | 2x10^-53^ | 83.84% | KAF5790247.1 |
| polyribonucleotide nucleotidyltransferase [*Tanacetum cinerariifolium*] | *Tanacetum cinerariifolium* | 84% | 1x10^-41^ | 76.60% | GEU52338.1 |
| Nucleotide BLAST | | | | | |
| *Arabidopsis thaliana* argininosuccinate lyase (AT5G02580), mRNA | *Arabidopsis thaliana* | 52% | 3x10^-26^ | 77.19% | NM_120336.4 |

Supplementary Table S3. List of light-responsive regulatory elements in *TkPEL-like* promotor sequences.

|  | Gene and organism | Transcription factor binding site | Binding factor | Position and sequence |
| --- | --- | --- | --- | --- |
| GWHGBCHF020456 (20 total) | *CHS*  *Petroselinum crispum* | ACE (CHS) | bZIP factors CPRF1+4 | - strand 787-778 CCACGTGaCC |
|  | *GRF1+2*  *Zea mays* | G-box | GBF | + strand 780-787 TCACGTGG |
|  | *Adh*  *Arabidopsis thaliana* | G-box | GBF3 | + strand 779-788 GtCACGTGGA |
|  | *Patatin*  *Solanum tuberosum* | G-box | GBF | - strand 788-779 TCCACGTGaC |
|  | *Cab1R*  *Oryza sativa* | G-box | Unknown nuclear factor | - strand 804-796 TATGTGGCA |
|  | *Synthetic oligonucleotide* | G-box | AtbZIP1 | - strand 787-778 CCACGTGaCC |
|  | *PSY*  *Arabidopsis thaliana* | G-box | PIF1 | - strand 788-779 TCCACGTGaC |
|  | *ANAC019/ ANAC055*  *Arabidopsis thaliana* | PIF7 BS1/2 | PIF7 | + strand 779-788 GtCACGTGGA |
| GWHGBCHF024124 (21 total) | *CHS*  *Petroselinum crispum* | Box II/  ACE (CHS) | bZIP factors CPRF1-3/  CPRF1+4 | - strand 785-776  CCACGTGaCC |
|  | *Aux28*  *Glycine max* | B1-core | SGBF-1+2 | - strand 786-777  TCCACGTGaC |
|  | *GRF1+2*  *Zea mays* | G-box | GBF | + strand 778-785 TCACGTGG |
|  | *Adh*  *Arabidopsis thaliana* | G-box | GBF3 | + strand 777-786 GtCACGTGGA |
|  | *Patatin*  *Solanum tuberosum* | G-box | GBF | - strand 786-777 TCCACGTGaC |
|  | *IAA17/AXR3*  *Arabidopsis thaliana* | G-box | STF1/HY5 | - strand 787-776  ATCCACGTGaCc |
|  | *Cab1R*  *Oryza sativa* | G-box | Unknown nuclear factor | - strand 802-794  TATGTGGCA |
|  | *Synthetic oligonucleotide* | G-box | AtbZIP1 | - strand 785-776 CCACGTGaCC |
|  | *PSY*  *Arabidopsis thaliana* | G-box | PIF1 | - strand 786-777  TCCACGTGaC |
|  | *ANAC019/ ANAC055*  *Arabidopsis thaliana* | PIF7 BS1/2 | PIF7 | + strand 777-786 GtCACGTGGA |
|  | *ANAC072*  *Arabidopsis thaliana* | IF7 BS2 | CBF1-4 | + strand 777-786  GtCACGTGGA |

Supplementary Table S4. Chlorophyll concentrations in *N. benthamiana* leaves expressing *TkPEL-like*.

Supplementary Table S5. Chlorophyll concentrations in leaves of pQ35S::*TkPEL-like T. koksaghyz* and NIL controls.

Supplementary Table S6. Quantification of pentacyclic triterpenoids and precursor molecules in pQ35S::*TkPEL-like T. koksaghyz* and NIL controls. RI = retention indices.

Supplementary Table S7. List of DEGs related to photosynthesis, light regulation and circadian rhythm. RNA and Protein IDs refer to the *T. koksaghyz* genome (Lin *et al.*, 2022).

| RNA ID | Protein ID | Short Name/  KEGG Annotation | Annotation NCBI/UniProt | Log_2_(FC) |
| --- | --- | --- | --- | --- |
| Photosynthesis-related genes | | | | |
| GWHTBCHF006034 | GWHPBCHF007309 | LHCB1 | light-harvesting complex II chlorophyll a/b binding protein 1 | 2.07 |
| GWHTBCHF021673 | GWHPBCHF025780 | LHCB1 | light-harvesting complex II chlorophyll a/b binding protein 1 | 1.5 |
| GWHTBCHF006036 | GWHPBCHF007311 | LHCB1 | light-harvesting complex II chlorophyll a/b binding protein 1 | 2.98 |
| GWHTBCHF021674 | GWHPBCHF025781 | LHCB1 | light-harvesting complex II chlorophyll a/b binding protein 1 | 1.5 |
| GWHTBCHF021672 | GWHPBCHF025779 | LHCB1 | light-harvesting complex II chlorophyll a/b binding protein 1 | 1.5 |
| GWHTBCHF038275 | GWHPBCHF045569 | LHCB1 | light-harvesting complex II chlorophyll a/b binding protein 1 | 6.92 |
| GWHTBCHF030128 | GWHPBCHF035844 | psbA | photosystem II P680 reaction center D1 protein | 1.22 |
| GWHTBCHF000164 | GWHPBCHF000184 | psbA | photosystem II P680 reaction center D1 protein | 1.07 |
| GWHTBCHF000178 | GWHPBCHF000198 | psbA | photosystem II P680 reaction center D1 protein | 1.29 |
| GWHTBCHF022904 | GWHPBCHF027252 | psbW | photosystem II PsbW protein | 1.6 |
| GWHTBCHF015567 | GWHPBCHF018544 | psaF | photosystem I subunit III | -4.29 |
| GWHTBCHF044723 | GWHPBCHF053248 | psaH | photosystem I subunit VI | 2.21 |
| GWHTBCHF028625 | GWHPBCHF034008 | psaL | photosystem I subunit XI | 1.65 |
| GWHTBCHF027287 | GWHPBCHF032451 | psaO | photosystem I subunit PsaO | 1.27 |
| GWHTBCHF000832 | GWHPBCHF000967 | petC | cytochrome b6-f complex iron-sulfur subunit | -2.96 |
| GWHTBCHF026283 | GWHPBCHF031253 | petC | cytochrome b6-f complex iron-sulfur subunit | -3.46 |
| GWHTBCHF014120 | GWHPBCHF016839 | atpC | F-type H+-transporting ATPase subunit epsilon | 1.98 |
| GWHTBCHF014125 | GWHPBCHF016844 | atpC | F-type H+-transporting ATPase subunit epsilon | 4.03 |
| GWHTBCHF014119 | GWHPBCHF016838 | atpC | F-type H+-transporting ATPase subunit epsilon | 1.98 |
| GWHTBCHF000256 | GWHPBCHF000283 | atpF | F-type H+-transporting ATPase subunit b | 1.55 |
| Light regulation-related genes | | | | |
| GWHTBCHF013774 | GWHPBCHF016433 | COP1 | E3 ubiquitin-protein ligase | 1.36 |
| GWHTBCHF043396 | GWHPBCHF051642 | COP1 | E3 ubiquitin-protein ligase | 1.74 |
| GWHTBCHF007304 | GWHPBCHF008823 | PIF3 | phytochrome-interacting factor 3 | 1.08 |
| GWHTBCHF040634 | GWHPBCHF048373 | PIF1 | ATP-dependent DNA helicase PIF1-like [Cynara cardunculus var. scolymus] | -4.67 |
| GWHTBCHF003202 | GWHPBCHF003845 | PIF1 | transcription factor PIF1 isoform X4 [Lactuca sativa] | 1.21 |
| GWHTBCHF002418 | GWHPBCHF002909 | PIF1 | ATP-dependent DNA helicase pif1-like [Lactuca sativa] | 2.13 |
| GWHTBCHF003026 | GWHPBCHF003647 | UVR8 | Ultraviolet-B receptor UVR8 (Protein UV-B RESISTANCE 8) (RCC1 domain-containing protein UVR8) | 4.12 |
| GWHTBCHF041835 | GWHPBCHF049760 | CSN subunit | COP9 signalosome complex subunit 2, Signalosome subunit 2 (Protein FUSCA 12) | 2.06 |
| GWHTBCHF021753 | GWHPBCHF025872 | FRS | Protein FAR1-RELATED SEQUENCE 8 | 6.50 |
| GWHTBCHF019705 | GWHPBCHF023481 | FRS | protein FAR1-RELATED SEQUENCE 5-like [Lactuca sativa] | 5.33 |
| GWHTBCHF042091 | GWHPBCHF050059 | FRS | Protein FAR1-RELATED SEQUENCE 6 | 3.81 |
| GWHTBCHF029804 | GWHPBCHF035452 | FRS | Protein FAR1-RELATED SEQUENCE 5 | 2.59 |
| GWHTBCHF034055 | GWHPBCHF040501 | FRS | protein FAR1-RELATED SEQUENCE 5-like [Lactuca sativa] | 2.53 |
| GWHTBCHF034703 | GWHPBCHF041264 | FRS | Putative protein FAR1-RELATED SEQUENCE 10 | 2.08 |
| GWHTBCHF016055 | GWHPBCHF019107 | FRS | Protein FAR1-RELATED SEQUENCE 8 | 1.54 |
| GWHTBCHF007026 | GWHPBCHF008482 | FRS | Putative protein FAR1-RELATED SEQUENCE 10 | -1.82 |
| GWHTBCHF007027 | GWHPBCHF008483 | FRS | Putative protein FAR1-RELATED SEQUENCE 10 | -1.82 |
| GWHTBCHF007028 | GWHPBCHF008484 | FRS | Putative protein FAR1-RELATED SEQUENCE 10 | -1.82 |
| Circadian rhythm-related genes | | | | |
| GWHTBCHF014016 | GWHPBCHF016717 | ELF3 | Protein EARLY FLOWERING 3 (Nematode-responsive protein) | 4.90 |
| GWHTBCHF031356 | GWHPBCHF037314 | PRR5 | Two-component response regulator-like APRR5 (Pseudo-response regulator 5) | 1.32 |
| GWHTBCHF014974 | GWHPBCHF017827 | PRR7 | Two-component response regulator-like APRR7 (Pseudo-response regulator 7) | 1.21 |
| GWHTBCHF014973 | GWHPBCHF017826 | PRR7 | Two-component response regulator-like APRR7 (Pseudo-response regulator 7) | 1.21 |
| GWHTBCHF016611 | GWHPBCHF019746 | CO | Zinc finger protein CONSTANS-LIKE 2 | 5.56 |
| GWHTBCHF034881 | GWHPBCHF041482 | PAP1, MYB75 | transcription factor MYB1 [Lactuca sativa] | -2.68 |
| GWHTBCHF034877 | GWHPBCHF041478 | PAP1, MYB75 | transcription factor MYB114 [Lactuca sativa] | 1.73 |
| GWHTBCHF027084 | GWHPBCHF032201 | FT | Protein HEADING DATE 3A (FT-like protein A) | 4.13 |
| GWHTBCHF042479 | GWHPBCHF050552 | CK2α | Casein kinase II subunit alpha-1, CK II, EC 2.7.11.1 (Casein kinase alpha 1, AtCKA1) | 3.72 |
| GWHTBCHF018716 | GWHPBCHF022296 | CDF1 | Cyclic dof factor 1 (Dof zinc finger protein DOF5.5, AtDOF5.5) | 1.48 |
| GWHTBCHF012568 | GWHPBCHF015017 | CHS | Chalcone synthase J, EC 2.3.1.74 (Naringenin-chalcone synthase J) | -5.16 |
| GWHTBCHF013633 | GWHPBCHF016276 | CHS | Chalcone synthase, EC 2.3.1.74 (Naringenin-chalcone synthase) | -2.17 |
| GWHTBCHF012567 | GWHPBCHF015016 | CHS | Chalcone synthase J, EC 2.3.1.74 (Naringenin-chalcone synthase J) | -4.22 |
| GWHTBCHF034809 | GWHPBCHF041396 | CHS | Chalcone synthase 3, EC 2.3.1.74 (Naringenin-chalcone synthase 3) | -3.60 |

Supplementary Table S8. List of DEGs related to RNA degradation and surveillance. RNA and Protein IDs refer to the *T. koksaghyz* genome (Lin *et al.*, 2022).

| RNA ID | Protein ID | Short Name/  KEGG Annotation | Annotation NCBI/UniProt | Log_2_(FC) |
| --- | --- | --- | --- | --- |
| GWHTBCHF020478 | GWHPBCHF024394 | PFK | ATP-dependent 6-phosphofructokinase 2, ATP-PFK 2, Phosphofructokinase 2, EC 2.7.1.11 (Phosphohexokinase 2) | 4.89 |
| GWHTBCHF005791 | GWHPBCHF007014 | ENO | Alpha-enolase, EC 4.2.1.11 (2-phospho-D-glycerate hydro-lyase) (Enolase 1) (Non-neural enolase, NNE) | -3.71 |
| GWHTBCHF015745 | GWHPBCHF018747 | ENO | Enolase, EC 4.2.1.11 (2-phospho-D-glycerate hydro-lyase) (2-phosphoglycerate dehydratase) (OSE1) | 1.18 |
| GWHTBCHF015743 | GWHPBCHF018745 | ENO | Enolase, EC 4.2.1.11 (2-phospho-D-glycerate hydro-lyase) (2-phosphoglycerate dehydratase) (OSE1) | 1.18 |
| GWHTBCHF015747 | GWHPBCHF018749 | ENO | Enolase, EC 4.2.1.11 (2-phospho-D-glycerate hydro-lyase) (2-phosphoglycerate dehydratase) (OSE1) | 1.18 |
| GWHTBCHF028947 | GWHPBCHF034402 | ENO | Cytosolic enolase 3, EC 4.2.1.11 (2-phospho-D-glycerate hydro-lyase 3) (2-phosphoglycerate dehydratase 3) | 1.30 |
| GWHTBCHF045338 | GWHPBCHF053972 | ENO | Enolase, EC 4.2.1.11 (2-phospho-D-glycerate hydro-lyase) (2-phosphoglycerate dehydratase) | -1.66 |
| GWHTBCHF015744 | GWHPBCHF018746 | ENO | Enolase, EC 4.2.1.11 (2-phospho-D-glycerate hydro-lyase) (2-phosphoglycerate dehydratase) (OSE1) | 1.18 |
| GWHTBCHF045476 | GWHPBCHF054141 | ENO | Enolase, EC 4.2.1.11 (2-phospho-D-glycerate hydro-lyase) (2-phosphoglycerate dehydratase) | -2.11 |
| GWHTBCHF015746 | GWHPBCHF018748 | ENO | Enolase, EC 4.2.1.11 (2-phospho-D-glycerate hydro-lyase) (2-phosphoglycerate dehydratase) (OSE1) | 1.18 |
| GWHTBCHF015748 | GWHPBCHF018750 | ENO | Enolase, EC 4.2.1.11 (2-phospho-D-glycerate hydro-lyase) (2-phosphoglycerate dehydratase) (OSE1) | 1.18 |
| GWHTBCHF004133 | GWHPBCHF004964 | MTR4 | LINE-1 reverse transcriptase homolog, EC 2.7.7.49 | 3.77 |
| GWHTBCHF018687 | GWHPBCHF022256 | PABPC | Polyadenylate-binding protein 2, PABP-2, Poly(A)-binding protein 2 | -1.17 |
| GWHTBCHF039425 | GWHPBCHF046925 | CNOT1 | Probable receptor-like protein kinase At1g30570, EC 2.7.11.- | -1.02 |
| GWHTBCHF046362 | GWHPBCHF055197 | CNOT7_8 | CCR4-NOT transcription complex subunit 1 (CCR4-associated factor 1) | 1.68 |
| GWHTBCHF033384 | GWHPBCHF039719 | CNOT7_8 | Probable CCR4-associated factor 1 homolog 11, EC 3.1.13.4 | 1.17 |
| GWHTBCHF026587 | GWHPBCHF031626 | DIS3/RRP44 | exosome complex exonuclease RRP44 homolog A-like [Prosopis alba] | 2.10 |
| GWHTBCHF008513 | GWHPBCHF010244 | DIS3/RRP44 | exosome complex exonuclease RRP44 homolog A-like [Prosopis alba] | 2.07 |
| GWHTBCHF026587 | GWHPBCHF031626 | RRP6/  EXOSC10 | exosome complex exonuclease RRP44 homolog A-like [Prosopis alba] | 2.10 |
| GWHTBCHF008513 | GWHPBCHF010244 | RRP6/  EXOSC10 | exosome complex exonuclease RRP44 homolog A-like [Prosopis alba] | 2.07 |
| GWHTBCHF032040 | GWHPBCHF038106 | RRP6/  EXOSC10 | Phosphoserine aminotransferase 2, chloroplastic, AtPSAT2, EC 2.6.1.52 | -1.15 |
| GWHTBCHF009710 | GWHPBCHF011642 | recQ | ATP-dependent DNA helicase Q-like 4A isoform X1 [Lactuca sativa] | 1.48 |
| GWHTBCHF026212 | GWHPBCHF031171 | recQ | ATP-dependent DNA helicase Q-like 4A isoform X1 [Lactuca sativa] | 2.52 |
| GWHTBCHF009367 | GWHPBCHF011244 | groEL | Chaperonin CPN60-like 2, mitochondrial (HSP60-like 2) | 1.0 |
| GWHTBCHF020831 | GWHPBCHF024798 | groEL | Chaperonin CPN60, mitochondrial (HSP60) | -1.84 |
| GWHTBCHF036056 | GWHPBCHF042875 | ERF1 | eukaryotic peptide chain release factor subunit 1-3 [Lactuca sativa] | 2.21 |
| GWHTBCHF036055 | GWHPBCHF042874 | ERF1 | eukaryotic peptide chain release factor subunit 1-3 [Lactuca sativa] | 2.21 |
| GWHTBCHF016166 | GWHPBCHF019240 | GLE1 | protein GLE1-like isoform X2 [Cynara cardunculus var. scolymus] | 2.99 |
| GWHTBCHF018995 | GWHPBCHF022635 | PAP | nuclear poly(A) polymerase 4 isoform X2 [Lactuca sativa] | 1.24 |
| GWHTBCHF018996 | GWHPBCHF022636 | PAP | nuclear poly(A) polymerase 4 isoform X2 [Lactuca sativa] | 1.24 |
| GWHTBCHF018997 | GWHPBCHF022637 | PAP | nuclear poly(A) polymerase 4 isoform X2 [Lactuca sativa] | 1.24 |
| GWHTBCHF017785 | GWHPBCHF021147 | CPSF1 | cleavage and polyadenylation specificity factor subunit 1 [Lactuca sativa] | 1.57 |
| GWHTBCHF017790 | GWHPBCHF021152 | CPSF1 | cleavage and polyadenylation specificity factor subunit 1 [Lactuca sativa] | 2.07 |
| GWHTBCHF016304 | GWHPBCHF019392 | SYMPK | symplekin isoform X2 [Lactuca sativa] | -1.88 |

Supplementary Table S9. List of DEGs of chlorophyll, carotenoid and precursor biosynthesis and connected pathways. RNA and Protein IDs refer to the *T. koksaghyz* genome (Lin *et al.*, 2022).

| RNA ID | Protein ID | Short Name/KEGG Annotation | No. | Log_2_(FC) |
| --- | --- | --- | --- | --- |
| GWHTBCHF014744 | GWHPBCHF017563 | HMGCR, hydroxymethylglutaryl-CoA reductase | 5 | 5.50 |
| GWHTBCHF029527 | GWHPBCHF035101 | HMGCR, hydroxymethylglutaryl-CoA reductase | 3 | 3.58 |
| GWHTBCHF029533 | GWHPBCHF035107 | HMGCR, hydroxymethylglutaryl-CoA reductase | 9 | 3.56 |
| GWHTBCHF029534 | GWHPBCHF035108 | HMGCR, hydroxymethylglutaryl-CoA reductase | 8 | 2.03 |
| GWHTBCHF029535 | GWHPBCHF035109 | HMGCR, hydroxymethylglutaryl-CoA reductase | 7 | 4.2 |
| GWHTBCHF038906 | GWHPBCHF046327 | DXS, 1-deoxy-d-xylulose-5-phosphate synthase | 7 | 1.04 |
| GWHTBCHF019930 | GWHPBCHF023750 | DXR, 1-deoxy-d-xylulose-5-phosphate reductoisomerase | 2 | -2.65 |
| GWHTBCHF001710 | GWHPBCHF002066 | IspH, 4-hydroxy-3-methylbut-2-en-1-yl diphosphate reductase | 3.1 | 7.31 |
| GWHTBCHF038972 | GWHPBCHF046403 | IspH, 4-hydroxy-3-methylbut-2-en-1-yl diphosphate reductase | 3.2 | 6.76 |
| GWHTBCHF003293 | GWHPBCHF003954 | ISPS, isoprene synthase | 1.1 | 1.52 |
| GWHTBCHF003294 | GWHPBCHF003955 | ISPS, isoprene synthase | 1.2 | 1.52 |
| GWHTBCHF016034 | GWHPBCHF019080 | FPPS, farnesyldiphosphate synthase | 2.1 | -1.23 |
| GWHTBCHF025874 | GWHPBCHF030773 | FPPS, farnesyldiphosphate synthase | 2.2 | -1.25 |
| GWHTBCHF025872 | GWHPBCHF030771 | FPPS, farnesyldiphosphate synthase | 2.3 | -1.25 |
| GWHTBCHF016036 | GWHPBCHF019082 | FPPS, farnesyldiphosphate synthase | 2.4 | -1.23 |
| GWHTBCHF025873 | GWHPBCHF030772 | FPPS, farnesyldiphosphate synthase | 2.5 | -1.25 |
| GWHTBCHF025870 | GWHPBCHF030769 | FPPS, farnesyldiphosphate synthase | 2.6 | -1.25 |
| GWHTBCHF016033 | GWHPBCHF019079 | FPPS, farnesyldiphosphate synthase | 2.7 | -1.23 |
| GWHTBCHF016035 | GWHPBCHF019081 | FPPS, farnesyldiphosphate synthase | 2.8 | -1.23 |
| GWHTBCHF025871 | GWHPBCHF030770 | FPPS, farnesyldiphosphate synthase | 2.9 | -1.25 |
| GWHTBCHF034449 | GWHPBCHF040951 | GGPS, geranylgeranyldiphosphate synthase | 7 | -4.68 |
| GWHTBCHF010356 | GWHPBCHF012414 | GGPS, geranylgeranyldiphosphate synthase | 5 | 1.10 |
| GWHTBCHF024114 | GWHPBCHF028673 | CRTISO, prolycopene isomerase | 1 | -3.55 |
| GWHTBCHF009162 | GWHPBCHF011006 | ZEP, zeaxanthin epoxidase | 1.1 | -2.42 |
| GWHTBCHF045167 | GWHPBCHF053775 | ZEP, zeaxanthin epoxidase | 1.2 | -1.27 |
| GWHTBCHF002200 | GWHPBCHF002653 | NCED, 9-*cis*-epoxycarotenoid dioxygenase | 1 | 1.5 |
| GWHTBCHF006620 | GWHPBCHF008002 | NCED, 9-*cis*-epoxycarotenoid dioxygenase | 2 | 2.56 |
| GWHTBCHF026592 | GWHPBCHF031632 | NCED, 9-*cis*-epoxycarotenoid dioxygenase | 3 | 3.41 |
| GWHTBCHF011688 | GWHPBCHF014018 | NCED, 9-*cis*-epoxycarotenoid dioxygenase | 4 | 5.87 |
| GWHTBCHF023747 | GWHPBCHF028235 | chlH, Mg chelatase subunit H | 1.1 | 1.33 |
| GWHTBCHF023748 | GWHPBCHF028238 | chlH, Mg chelatase subunit H | 1.2 | 5.19 |
| GWHTBCHF002552 | GWHPBCHF003086 | chlE, Mg-protoporphyrin IX monomethyl ester cyclase | 1.1 | 1.63 |
| GWHTBCHF031847 | GWHPBCHF037894 | chlE, Mg-protoporphyrin IX monomethyl ester cyclase | 1.2 | 2.38 |
| GWHTBCHF041876 | GWHPBCHF049808 | POR, protochlorophyllide reductase | 1 | 4.09 |
| GWHTBCHF018730 | GWHPBCHF022314 | GGPR, geranylgeranyldiphosphate reductase | 1 | 3.54 |
| GWHTBCHF020317 | GWHPBCHF024203 | SQS, squalene synthase | 1 | 1.44 |
| GWHTBCHF040962 | GWHPBCHF048767 | SQLE, squalene monooxygenase | 2.1 | -1.2 |
| GWHTBCHF040964 | GWHPBCHF048769 | SQLE, squalene monooxygenase | 2.2 | -1.2 |
| GWHTBCHF040963 | GWHPBCHF048768 | SQLE, squalene monooxygenase | 2.3 | -1.2 |
| GWHTBCHF040965 | GWHPBCHF048770 | SQLE, squalene monooxygenase | 2.4 | -1.2 |
| GWHTBCHF013441 | GWHPBCHF016046 | *cis*PT, *cis*-prenyltransferase | 2 | 1.01 |
| GWHTBCHF006764 | GWHPBCHF008169 | *cis*PT, *cis*-prenyltransferase*-like* | L1 | 1.01 |
| GWHTBCHF013439 | GWHPBCHF016044 | *cis*PT, *cis*-prenyltransferase | 1.1 | 1.01 |
| GWHTBCHF013440 | GWHPBCHF016045 | *cis*PT, *cis*-prenyltransferase | 1.2 | 1.01 |
| GWHTBCHF023398 | GWHPBCHF027821 | *cis*PT, *cis*-prenyltransferase | 8 | 1.44 |
| GWHTBCHF023400 | GWHPBCHF027823 | *cis*PT, *cis*-prenyltransferase | 7 | 1.65 |
| GWHTBCHF029038 | GWHPBCHF034521 | *cis*PT, *cis*-prenyltransferase | 3 | 2 |
